# Cytotoxic Thiodiketopiperazine Derivatives from the Deep Sea-Derived Fungus *Epicoccum nigrum* SD-388

**DOI:** 10.3390/md18030160

**Published:** 2020-03-13

**Authors:** Lu-Ping Chi, Xiao-Ming Li, Li Li, Xin Li, Bin-Gui Wang

**Affiliations:** 1 Key Laboratory of Experimental Marine Biology, Institute of Oceanology, Chinese Academy of Sciences, Nanhai Road 7, Qingdao 266071, China; chiluping@qdio.ac.cn (L.-P.C.); lixmqdio@126.com (X.-M.L.); 15738367975@163.com (L.L.); 2 Laboratory of Marine Biology and Biotechnology, Qingdao National Laboratory for Marine Science and Technology, Wenhai Road 1, Qingdao 266237, China; 3 University of Chinese Academy of Sciences, Yuquan Road 19A, Beijing 100049, China; 4 Center for Ocean Mega-Science, Chinese Academy of Sciences, Nanhai Road 7, Qingdao 266071, China

**Keywords:** *Epicoccum nigrum*, deep-sea-derived fungus, thiodiketopiperazines, diketopiperazine enantiomers, cytotocxic activity

## Abstract

Four new thiodiketopiperazine alkaloids, namely, 5’-hydroxy-6’-ene-epicoccin G (**1**), 7-methoxy-7’-hydroxyepicoccin G (**2**), 8’-acetoxyepicoccin D (**3**), and 7’-demethoxyrostratin C (**4**), as well as a pair of new enantiomeric diketopiperazines, (±)-5-hydroxydiphenylalazine A (**5**), along with five known analogues (**6**–**10**), were isolated and identified from the culture extract of *Epicoccum nigrum* SD-388, a fungus obtained from deep-sea sediments (−4500 m). Their structures were established on the basis of detailed interpretation of the NMR spectroscopic and mass spectrometric data. X-ray crystallographic analysis confirmed the structures and established the absolute configurations of compounds **1**–**3**, while the absolute configurations for compounds **4** and **5** were determined by ECD calculations. Compounds **4** and **10** showed potent activity against Huh7.5 liver tumor cells, which were comparable to that of the positive control, sorafenib, and the disulfide bridge at C-2/C-2’ is likely essential for the activity.

## 1. Introduction

Natural products have historically been a rich source of new drugs or drug candidates. Strikingly, deep-sea-derived microorganisms survive under extreme environments, leading to special biological diversity and prolific metabolisms differing from those of terrestrial microorganisms. Recently, deep-sea-sourced microbial natural products have been reported with high hit-rates from bioactivity screening, particularly in the antitumor area [1,2].

*Epicoccum nigrum* is a chemically distinct fungal species with potential to produce structurally unique secondary metabolites including thiodiketopiperazines (TDKPs) [3,4], polyketides [5], and polysaccharides [6]. Some of these metabolites exhibited intriguing biological properties, such as antimicrobial [3], cytotoxic [4], and antioxidant activities [5,6].

The TDKP derivatives are a family of diketopiperazines which have been isolated from several fungal sources, such as *Epicoccum nigrum* [3], *Exserohilum rostratum* [7], *Penicillium brocae* [8], and *Penicillium adametzioides* [9].

**Figure 1:**
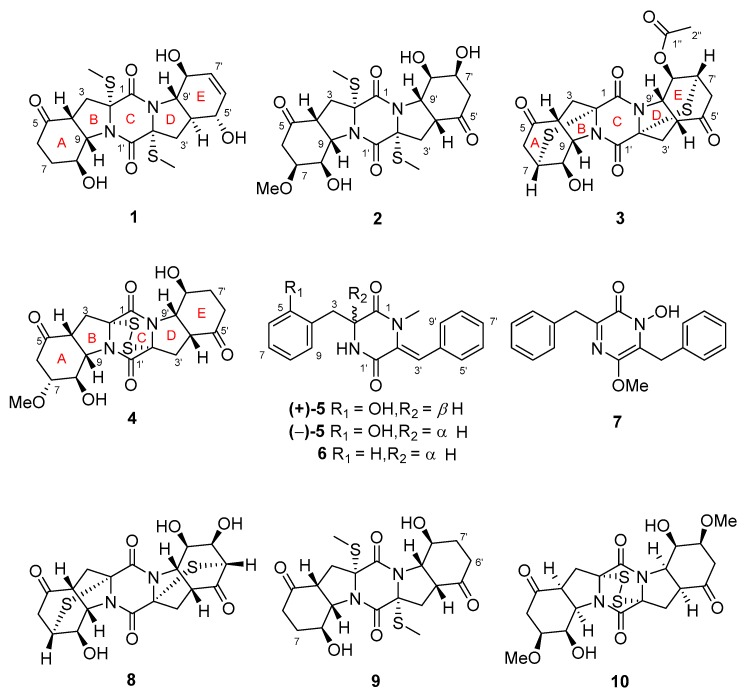
Structures of the isolated compounds **1**–**10.**

In continuation of our research aimed at discovery of bioactive metabolites from marine-derived microorganisms [10,11,12], a fungal strain of *Epicoccum nigrum* SD-388 was isolated from a deep-sea sediment sample collected at a depth of 4500 m. Chemical investigation of the fungus resulted in the isolation of spiroepicoccin A, an unusual spiro-TDKP derivative, whose stereochemistry could not be elucidated by conventional NMR methods and was solved based on residual chemical shift anisotropies [12]. This result encouraged us to perform a further study of the fungus and has led to the isolation of four new TDKPs including 5’-hydroxy-6’-ene-epicoccin G (**1**), 7-methoxy-7’-hydroxyepicoccin G (**2**), 8’-acetoxyepicoccin D (**3**), and 7’-demethoxyrostratin C (**4**), as well as a pair of new enantiomeric diketopiperazines (DKPs), (±)-5-hydroxydiphenylalazine A ((±)-**5**), together with five known analogues including diphenylalazine A (**6**) [4], emeheterone (**7**) [13], epicoccins E (**8**) and G (**9**) [4], and rostratin C (**10**) [7] (Figure 1). Details of the isolation and purification, structural elucidation, and cytotoxic potency against Huh7.5 liver tumor cells of compounds **1**–**10** are described herein.

## 2. Results and Discussion

### 2.1. Structure Elucidation of the New Compounds

The fungal strain *E. nigrum* SD-388 was cultured on the rice solid medium, which was further exhaustively extracted with EtOAc to afford an extract. Fractionation of the extracts by a combination of column chromatography (CC) over silica gel, Lobar LiChroprepRP-18, SephadexLH-20, as well as semi-preparative HPLC, yielded compounds **1**–**10**.

**Figure 2:**
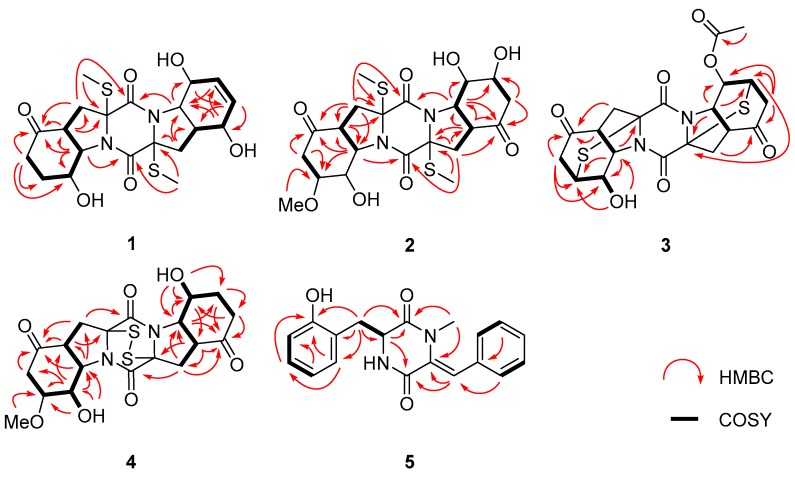
Key ^1^H-^1^H COSY (bold lines) and HMBC (red arrows) correlations of compounds **1**–**5**.

Compound **1**, initially obtained as colorless gum, gave a pseudomolecular ion peak at *m/z* 455.1293 [M + H]^+^ by HR-ESI-MS, consistent with a molecular formula of C_20_H_26_N_2_O_6_S_2_, indicating 9 degrees of unsaturation. The ^1^H-, ^13^C-NMR, and DEPT spectroscopic data (Table 1 and Table 2) revealed the presence of two methyls, four sp^3^ hybridized methylenes, nine methines (with five oxygenated/nitrogenated and two olefinic), five nonprotonated carbons (with one ketone and two amide carbonyls), as well as three exchangeable protons. Detailed analysis of the NMR data disclosed that the structure of **1** was similar to that of epicoccin G (**9**), a well described TDKP derivative identified from a *Cordyceps*-colonizing fungus *Epicoccum nigrum* XZC04-CS-302 in 2009 [4]. However, signals for two CH_2_ groups at *δ*_H_/*δ*_C_ 2.20 and 2.59/33.8 (C-6’) and at *δ*_H_/*δ*_C_ 1.88 and 2.12/25.8 (C-7’) in compound **9**, were replaced by two olefinic CH groups at *δ*_H_/*δ*_C_ 5.68/133.3 (C-6’) and *δ*_H_/*δ*_C_ 5.53/129.9 (C-7’) in the NMR spectra of **1**. Furthermore, the signal for the ketone group (C-5’) of **9** (*δ*_C_ 207.7) was replaced by an oxygenated methine (*δ*_H_/*δ*_C_ 4.11/71.3) in **1**. The COSY correlations for the spin system from H-3’ through H-9’ via H-4’~H-8’ and the HMBC correlations from H-5’ to C-7’ and C-9’, from H-6’ to C-4’ and C-5’, and from H-7’ to C-9’, confirmed the proposed structure of **1** (Figure 2).

**Figure 3:**
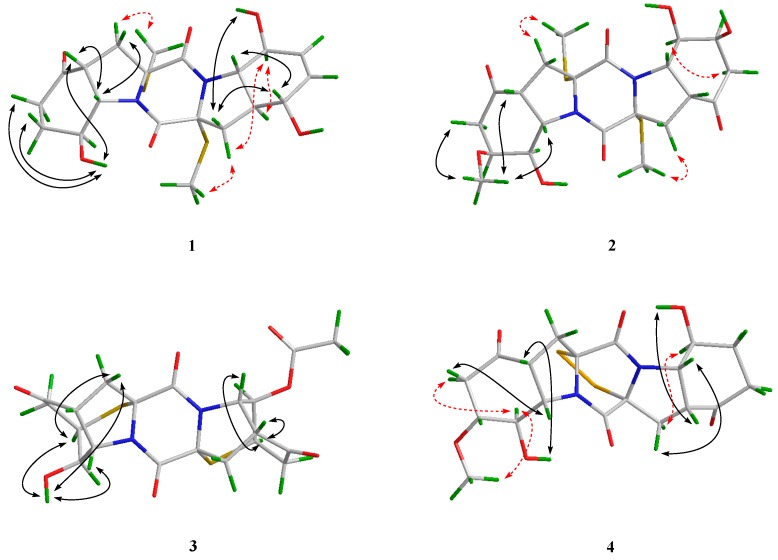
Key NOE correlations of compounds **1**–**4** (black solid lines: *β*-orientation; red dashed lines: *α*-orientation).

The relative configuration of **1** was deduced from analysis of the NOESY spectrum. NOE correlations from H-9 to H-3*β* and H-4, and from the proton of 8-OH to H-4, H-6*β*, and H-7*β*, indicated the cofacial orientation of these groups (Figure 3). Besides, NOEs from H-3*α* to 2-SMe placed them on another face, opposite to that of H-4, H-9, and 8-OH. Moreover, NOE cross-peaks from H-8’ to H-3’*α* and H-4’, and from H-3’*α* to 2’-SMe, confirmed them on the same spatial orientation, while NOE correlations from H-5’ to H-9’ and H-3’*β* placed these groups on the opposite face. On the basis of the above observation, the relative configurations for rings A/B and D/E were determined respectively. However, the relationship between these two units could not be correlated based on the NOESY experiment, because no diagnostic NOE cross-peak could be detected between rings A/B and D/E.

**Figure 4:**
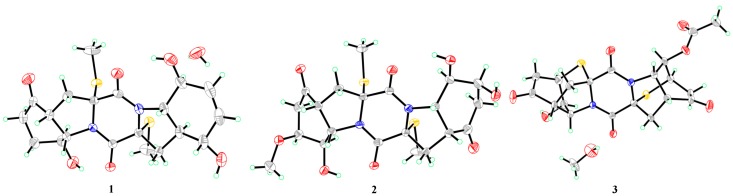
X-ray crystallographic structures of compounds **1***–***3**.

To fully assign the configuration of compound **1**, efforts toward a single crystal X-ray study were performed. By slow evaporation of the solvent (MeOH–H_2_O, 100:1) under refrigeration, quality crystals of **1** were obtained, making it feasible for an X-ray crystallographic experiment which confirmed not only the planar structure, but also the relative configuration of compound **1** (Figure 4). The defined Flack parameter 0.01(3) determined the absolute configuration of **1** as 2*R*, 4*R*, 8*S*, 9*S*, 2’*R*, 4’*S*, 5’*S*, 8’*S*, and 9’*S*, and the trivial name 5’-hydroxy-6’-ene-epicoccin G was assigned to compound **1**.

The elemental composition of **2** was established to be C_21_H_28_N_2_O_8_S_2_ by analysis of HR-ESI-MS and NMR data, indicating nine degrees of unsaturation. The ^1^H- and ^13^C-NMR data of **2** were similar to those of epicoccin G (**9**), a symmetrical TDKP derivative characterized from *E. nigrum* XZC04-CS-302 [4], except that the signals of two methylene groups at *δ*_H_/*δ*_C_ 1.88 and 2.12/25.8 (CH_2_-7 and CH_2_-7’) in **9** were replaced by two oxygenated methine groups at *δ*_H_/*δ*_C_ 3.82/75.8 (CH-7) and *δ*_H_/*δ*_C_ 4.12/65.7 (CH-7’) in **2**, respectively. Moreover, signals for a methoxy group at *δ*_H_/*δ*_C_ 3.25/55.8 (7-OMe) were also observed (Table 1 and Table 2). The methoxy group was assigned at C-7 based on the observed HMBC correlation from 7-OMe to C-7. Supported by key COSY correlations from H-6 to H-7, and from H-6’ to H-7’, as well as by HMBC correlations from H-6 and H-9 to C-7 and from H-6’ and H-9’ to C-7’ (Figure 2), the planar structure of compound **2** was determined.

The relative configuration of **2** was assigned by analysis of *J*-coupling constants and NOESY data. A coupling constant of 8.5 Hz between H-4 and H-9 as well as between H-4’ and H-9’ suggested their *cis* relationships, as reported in the previous literature [7]. NOE correlations from the proton of 7-OMe to H-4 and H-9 indicated the cofacial orientation of these groups. However, the relative configurations of **2** could not be fully assigned due to the lack of some key NOE correlations.

A single crystal of **2** was cultivated, after attempts by dissolving the samples in MeOH–H_2_O (100:1) followed by slow evaporation under refrigeration for two weeks. Once the Cu/K*α* X-ray crystallographic experiment was conducted (Figure 4), the structure and absolute configuration of **2** were assigned as 2*R*, 4*R*, 7*S*, 8*R*, 9*S*, 2’*R*, 4’*R*, 7’*S*, 8’*R*, and 9’*S*, with a Flack parameter of 0.02(4). Compound **2** was named 7-methoxy-7’-hydroxyepicoccin G.

The accurate mass data measured by HR-ESI-MS of compound **3** assigned its molecular formula, C_20_H_20_N_2_O_7_S_2_ (12 degrees of unsaturation), and was supported by the NMR data. The ^1^H- and ^13^C-NMR data of **3** (Table 1 and Table 2) showed close similarity to those of epicoccin D, a TDKP derivative isolated from the fungal strain *E. nigrum* (2203) in 2007 [3]. However, resonances for an ester carbonyl carbon (*δ*_C_ 168.8, C-1″) and a methyl group (*δ*_H_/*δ*_C_ 2.04/20.6, CH_3_-2″) were observed in the NMR spectra of **3**. Deshielded shift at *δ*_H_ 5.04 for H-8’ in **3** was detected, compared to that of *δ*_H_ 4.00 in epicoccin D. The above observation suggested that compound **3** was a C-8’ acetylated derivative of epicoccin D. The relative configuration of **3** was assigned on the basis of the NOESY experiment and *J*-coupling constants. For ring A of **3**, NOE correlations from the proton of OH-8 to H-7 and H-9 revealed the same orientation of these groups (Figure 3). In addition, the *cis* relationship between H-4 and H-9 was established by the coupling constant (*J* = 8.3 Hz) which is in agreement with that of rostratin B (*J* = 7.2 Hz) [7]. However, the relative configurations of ring E could not be solved as it lacked some key NOE correlations. To unequivocally determine the relative and absolute configurations, single crystals for **3** were cultivated upon slow evaporation of the solvent (MeOH) and a Cu/Kα X-ray diffraction analysis was conducted (Figure 4). The final refinement of the X-ray data resulted in a 0.02(3) Flack parameter, allowing for the assignment of the absolute configuration as 2*R*, 4*R*, 7*R*, 8*R*, 9*S*, 2’*R*, 4’*R*, 7’*R*, 8’*R*, and 9’*S*.

Compound **4** was initially isolated as a colorless powder. Its molecular formula was postulated as C_19_H_22_N_2_O_7_S_2_ through HR-ESI-MS analysis, indicating 10 degrees of unsaturation. The 1D NMR data of **4** were similar to those of rostratin C (**10**), a DKP derivative isolated from the marine-derived fungal strain *Exserohilum rostratum* CNK-630 [7], with the exception of the disappeared signals for the oxygenated methine (C-7’) and the methoxy group attached to C-7’. In contrast, signals for a methylene group at *δ*_H_ 1.62/1.89 and *δ*_C_ 25.4 (CH_2_-7’) were observed in the NMR spectra of **4** (Table 1 and Table 2), indicating that **4** is a 7’-demethoxy derivative of **10**. The 2D NMR correlations supported this inference by the COSY correlations from H-7*’* to H-6*’* and H-8*’*, and HMBC correlations from H-7*’* to C-5*’* and from H-9*’* to C-7*’* (Figure 2).

The relative configuration for rings A and E of **4** were determined by analysis of NOESY data. NOE correlations, with respect to ring A from H-8 to H-3*α* and 7-OMe, placed them on the same face. Meanwhile, NOEs from the proton of 8-OH to H-4, and from H-3*β* to H-9, disclosed the cofacial orientation of these groups. As for ring E, the coupling constant (*J* = 8.0 Hz) observed between H-4’ and H-9’ revealed their *cis* relationship [7]. In addition, NOEs from the proton of 8’-OH to H-4’, and from H-3’*β* to H-9’, revealed them on the cofacial orientation. Whereas NOE from H-3’*α* to H-8’ revealed that these groups were on the other face (Figure 3).

**Figure 5:**
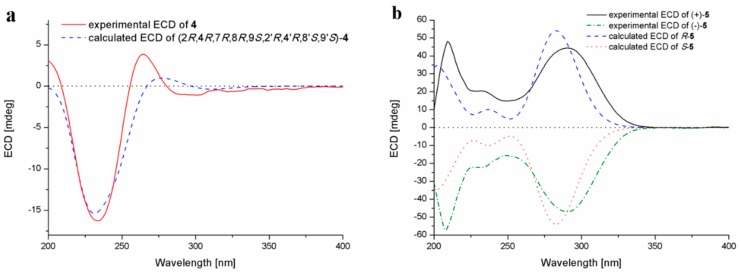
Experimental and calculated ECD spectra of compounds **4** (**a**) and **5** (**b**).

The assignment of the absolute configurations at C-2/C-2’ were established by analysis of ECD cotton effects (CEs) following the rules reported by the previous reference [14]. The ECD spectrum of **4** showed a positive CE near 265 nm, which was characteristic for the 2*R*/2’*R* configurations in TDKPs. The whole absolute configuration of **4** was further studied using the time-dependent density functional (TDDFT)-ECD calculation in Gaussian 09. The ECD spectra of four possible stereoisomers of **4**, including (2*R*, 4*R*, 7*R*, 8*R*, 9*S*, 2’*R*, 4’*R*, 8’*S*, 9’*S*)-**4**, (2*R*, 4*R*, 7*R*, 8*R*, 9*S*, 2’*R*, 4’*S*, 8’*R*, 9’*R*)-**4**, (2*R*, 4*S*, 7*S*, 8*S*, 9*R*, 2’*R*, 4’*R*, 8’*S*, 9’*S*)-**4**, and (2*R*, 4*S*, 7*S*, 8*S*, 9*R*, 2’*R*, 4’*S*, 8’*R*, 9’*R*)-**4**, were calculated. The experimental ECD spectrum for **4** showed agreement with that calculated for (2*R*, 4*R*, 7*R*, 8*R*, 9*S*, 2’*R*, 4’*R*, 8’*S*, 9’*S*)-**4** (Figure 5a), allowing the elucidation of whole chiral centers as 2*R*, 4*R*, 7*R*, 8*R*, 9*S*, 2’*R*, 4’*R*, 8’*S*, and 9’*S*.

Compound **5**, obtained as a yellow oil, was assigned the molecular formula C_19_H_18_N_2_O_3_ by HR-ESI-MS, and required 12 degrees of unsaturation. Analysis of the ^1^H- and ^13^C-NMR data (Table 1 and Table 2) revealed that compound **5** had same basic structure as that of the previously reported diphenylalazine A (**6**), which was identified from the fungus *E. nigrum* XZC04-CS-302 [4]. However, the aromatic methine at C-5 in **6** (*δ*_H_/*δ*_C_ 7.18/129.82) was replaced by a nonprotonated and hydroxylated carbon (*δ*_C_ 156.1) in **5**. HMBC correlations from H-3, H-7, and H-9 to C-5, supported this deduction. The planar structure of **5** was thus established as 5-hydroxydiphenylalazine A. However, the specific optical rotation value of ${\left[\alpha \right]}_{25}^{D}$ = 0 (c 0.10, MeOH) revealed the racemic nature of compound **5**, which was also confirmed by the fact that no cotton effects were observed in the ECD spectrum (Figure S36). Separation of **5** by HPLC using the Daicel Chiral-pak IC column yielded (+)-**5** and (–)-**5** (Figure S37), which were individually determined absolute configurations by experimental and calculated ECD spectra (Figure 5b), and assigned (+)-**5** as 2*R* and (–)-**5** as 2*S*. 

In addition to compounds **1**–**5**, five known analogues (**6**–**10**) were also isolated. By detailed spectroscopic analysis as well as comparison with reported data, the structures of compounds **6**–**10** were identified as diphenylalazines A (**6**) [4], emeheterone (**7**) [13], epicoccins E (**8**) and G (**9**) [4], and rostratin C (**10**) [7].

### 2.2. Biological Activities of the Isolated Compounds

**Figure 6:**
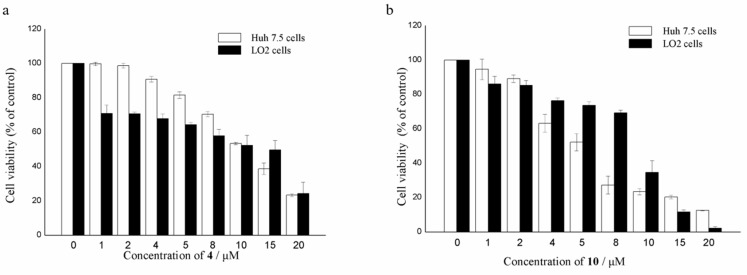
Cell viability of Huh7.5 liver cancer cells and LO2 normal liver cells treated with compounds **4** (**a**) and **10** (**b**).

All of the isolated compounds were assayed for their activities against Huh7.5 liver tumor cells. Among them, only compounds **4** and **10** displayed significant cytotoxic effects against Huh7.5 with cell viability less than 30% at the concentration of 20 μΜ. As shown in Figure 6, the growth-inhibiting effects of **4** and **10** were concentration-dependent, with IC_50_ values of 9.52 and 4.88 μM, respectively, which were comparable to that of the positive control, sorafenib (IC_50_ 8.2 μM). Compounds **4** and **10** were also measured for their cytotoxicity against human normal liver LO2 cell line. The results showed that compound **4** exhibited the inhibitory effects against normal liver cells, similar to that of cancer cells (Figure 6). However, compound **10** was less sensitive to normal liver cells than liver cancer cells, only at a narrow concentration range of 4~10 μM. The results suggested that the disulfide bridge at C-2/C-2’ is likely essential for the activity.

## 3. Experimental Section

### 3.1. General Experimental Procedures

Melting points were acquired through an SGW X-4 micro-melting-point apparatus (Shanghai Shenguang Instrument Co. Ltd, Shanghai, China). Optical rotations were measured using an Optical Activity AA-55 polarimeter (Optical Activity Ltd., Cambridgeshire, UK). UV spectra were determined by a PuXi TU-1810 UV-visible spectrophotometer (Shanghai Lengguang Technology Co. Ltd., Shanghai, China), and ECD spectra were obtained with a JASCO J-715 spectropolarimeter (JASCO, Tokyo, Japan). NMR spectra were recorded on a Bruker Avance 500 spectrometer (Bruker Biospin Group, Karlsruhe, Germany), using solvent chemical shifts (DMSO-*d*_6_: *δ*_H_/*δ*_C_ 2.50/39.52) as reference. HR-ESI-MS were measured on an API QSTAR Pulsar 1 mass spectrometer (Applied Biosystems, Foster, Waltham, MA, USA). Analytical and semi-preparative reversed-phase HPLC separations were performed by a Dionex HPLC system, equipped with P680 pump (Dionex, Sunnyvale, CA, USA), ASI-100 automated sample injector, and UVD340U multiple wavelength detector controlled by Chromeleon software (version 6.80). Commercially available Lobar LiChroprep RP-18 (40–63 μm, Merck, Darmstadt, Germany), Si gel (200–300 mesh, Qingdao Haiyang Chemical Co., Qingdao, China), and Sephadex LH-20 (Pharmacia, Pittsburgh, PA, USA) were used for column chromatography. Thin-layer chromatography (TLC) was carried out using precoated Si gel GF_254_ plates (Merck, Darmstadt, Germany). All solvents used were distilled prior to use.

### 3.2. Fungal Material

The fungal strain *Epicoccum nigrum* SD-388 was isolated from the deep-sea sediment collected in the West Pacific (depth 4500 m) on March 2015. The fungus was identified using a molecular biological protocol by DNA amplification and sequencing of the ITS (internal transcript spacer) region. The sequence data for the fungus have been deposited in GenBank with the accession no. MN089646. Through the BLAST searching, the fungus was identified as *Epicoccum nigrum* according to the ITS region sequence, which is the same (100%) as that of *E. nigrum* (accession no. KU254609). The strain is preserved at the Key Laboratory of Experimental Marine Biology, Institute of Oceanology, Chinese Academy of Sciences (IOCAS).

### 3.3. Fermentation

For chemical investigations, the fresh mycelia of the fungus were grown on PDA medium at 28 °C for one week and were then inoculated into 1 L Erlenmeyer flasks. The fungus was fermented statically at room temperature for 35 days in rice solid medium containing rice (70 g/flask), peptone (0.3%), yeast extract (0.5%), corn steep liquor (0.2%), monosodium glutamate (0.1%), Fe_2_(SO_4_)_3_ (0.002%), MgSO_4_·7H_2_O (0.07%), ZnSO_4_ (0.0001%), KH_2_PO_4_ (0.025%), and naturally sourced and filtered seawater (obtained from the Huiquan Gulf of the Yellow Sea near the campus of IOCAS, 100 mL/flask).

### 3.4. Extraction and Isolation

The fermented rice substrate was mechanically fragmented after incubation, and then extracted with petroleum ether (PE) to remove the low-polarity chemical constituents. The remaining culture was extracted thoroughly with EtOAc, which was filtered and evaporated under reduced pressure to give EtOAc extract (75.5 g).

The EtOAc extract was fractionated by Si gel VLC (vacuum liquid chromatography), using solvents of increasing polarity (PE-EtOAc, 20:1 to 1:1, and then CH_2_Cl_2_-MeOH, 20:1 to 1:1) to yield nine fractions (Frs. 1−9). Fr. 5 (6.6 g) was further separated by CC (Column Chromatography) over Lobar LiChroprep RP-18 with a MeOH-H_2_O gradient (from 1:9 to 10:0) to yield 10 subfractions (Frs. 5.1−5.10). Fr. 5.4 (453.8 mg) was subjected to CC on Si gel eluting with a CH_2_Cl_2_-MeOH gradient (from 500:1 to 200:1) to yield compounds **6** (187.6 mg) and **7** (15.5 mg). Fr. 6 (29.2 g) was repeatedly subjected to Si gel VLC and then fractionated by solvents of increasing polarity from CH_2_Cl_2_ to acetone to yield four subfractions (Frs. 6.1−6.4) based on HPLC and TLC analysis. Purification of Fr. 6.1 (6.2 g) by CC over Lobar LiChroprep RP-18 with a MeOH-H_2_O gradient (from 1:9 to 10:0) yielded 10 subfractions (Frs. 6.1.1−6.1.10). Fr. 6.1.1 (14.3 mg) was recrystallized from mixed solvents (MeOH-H_2_O, 10:1) to give **3** (6.7 mg). Fr. 6.1.2 (53.7 mg) was purified by prep-TLC and CC on Sephadex LH-20 (MeOH) to obtain **10** (34.1 mg). Fr. 6.1.3 (44.8 mg) was also recrystallized from MeOH to give **9** (21.9 mg). Fr. 6.1.4 (79.2 mg) was applied to semi-preparative HPLC (Elite ODS-BP column, 10 μm; 20 × 250 mm; 70% MeOH-H_2_O, 16 mL/min) to afford **4** (18.3 mg, *t*_R_ 29.6 min). Fr. 6.1. 5 (207.3 mg) was subjected to repeated CC on silica gel (CH_2_Cl_2_-MeOH, 140:1) and purified by prep-TLC and CC on Sephadex LH-20 (MeOH) to give **5** (9.4 mg). Fr. 6.2 (6.0 g) was split by CC over Lobar LiChroprep RP-18, silica gel, and Sephadex LH-20 to yield **2** (12.5 mg). Fr. 6.3 (6.5 g) was subjected to CC over Lobar LiChroprep RP-18, eluted with a MeOH-H_2_O gradient (from 1:9 to 10:0) to yield 10 subfractions (Frs. 6.3.1−6.3.10). Fr. 6.3.1 (32.1 mg) was recrystallized from mixed solvents (MeOH-H_2_O, 10:1) to afford **8** (13.7 mg). Fr. 6.3.3 (108.9 mg) was purified by semi-preparative HPLC (Elite ODS-BP column, 10 μm; 20 × 250 mm; 72% MeOH-H_2_O, 16 mL/min) to afford **1** (46.8 mg, *t*_R_ 31.2 min).

5’-Hydroxy-6’-ene-epicoccin G (**1**): Colorless cube crystal (MeOH-H_2_O); mp 161–163 °C; ${\left[\alpha \right]}_{25}^{D}$ −95.7 (*c* 0.23, MeOH); UV (MeOH) *λ*_max_ (log *ε*) 204 (3.99) nm; ECD (4.18 mM, MeOH) *λ*_max_ (*Δε*) 200 (−1.96), 233 (+2.46), 260 (−3.07) nm; ^1^H and ^13^C NMR data, Table 1 and Table 2; ESIMS *m/z* 455 [M + H]^+^; HRESIMS *m/z* 455.1293 [M + H]^+^ (calcd for C_20_H_27_O_6_N_2_S_2_, 455.1305).

7-Methoxy-7’-hydroxyepicoccin G (**2**): Colorless cube crystal (MeOH); mp 173–175 °C; ${\left[\alpha \right]}_{25}^{D}$ −138.9 (*c* 0.18, MeOH); UV (MeOH) *λ*_max_ (log *ε*) 204 (4.16) nm; ECD (4.80 mM, MeOH) *λ*_max_ (*Δε*) 200 (−3.35), 231 (+3.91), 259 (−2.53)nm; ^1^H and ^13^C NMR data, Table 1 and Table 2; ESIMS *m/z* 501 [M + H]^+^; HRESIMS *m/z* 501.1360 [M + H]^+^ (calcd for C_21_H_29_O_8_N_2_S_2_, 501.1360).

8’-Acetoxyepicoccin D (**3**): Colorless needle crystal (MeOH); mp 233–235 °C; ${\left[\alpha \right]}_{25}^{D}$ +225.6 (*c* 0.02, MeOH); UV (MeOH) *λ*_max_ (log *ε*) 204 (3.75) nm; ECD (2.15 mM, MeOH) *λ*_max_ (*Δε*) 218 (+1.24), 252 (+0.31), 290 (−0.10) nm; ^1^H and ^13^C NMR data, Table 1 and Table 2; ESIMS *m/z* 482 [M + NH_4_]^+^, *m/z* 487 [M + Na]^+^; HRESIMS *m/z* 482.1045 [M + NH_4_]^+^ (calcd for C_20_H_24_O_7_N_3_S_2_, 482.1050), 487.0601 [M + Na]^+^ (calcd for C_20_H_20_O_7_N_2_NaS_2_, 487.0604).

7’-Demethoxyrostratin C (**4**): Colorless amorphous powder; ${\left[\alpha \right]}_{25}^{D}$ −215.4 (*c* 0.13, MeOH); UV (MeOH) *λ*_max_ (log *ε*) 201 (4.06) nm; ECD (2.20 mM, MeOH) *λ*_max_ (*Δε*) 200 (+0.43), 234 (−2.24), 265 (+0.53) nm; ^1^H and ^13^C NMR data, Table 1 and Table 2; ESIMS *m/z* 455 [M + H]^+^; HRESIMS *m/z* 455.0930 [M + H]^+^ (calcd for C_19_H_23_O_7_N_2_S_2_, 455.0941).

(±)-5-Hydroxydiphenylalazine A (**5**): Yellow oil; UV (MeOH) *λ*_max_ (log *ε*) 200 (4.49) nm, 216 (4.19) nm, 283 (4.14) nm; ^1^H and ^13^C NMR data, Table 1 and Table 2; ESIMS *m/z* 323 [M + H]^+^; HRESIMS *m/z* 323.1383 [M + H]^+^ (calcd for C_19_H_19_O_3_N_2_, 323.1390).

(+)-**5**: ${\left[\alpha \right]}_{25}^{D}$ +350 (*c* 0.18, MeOH); ECD (5.59 mM, MeOH) *λ*_max_ (*Δε*) 209 (+2.59), 233 (+1.11), 291 (+2.41) nm.

(−)-**5**: ${\left[\alpha \right]}_{25}^{D}$ −345 (*c* 0.18, MeOH); ECD (5.59 mM, MeOH) *λ*_max_ (*Δε*) 206 (−3.11), 242 (−1.21), 288 (−2.55) nm.

### 3.5. X-Ray Crystallographic Analysis

Crystallographic data have been deposited in the Cambridge Crystallographic Data Centre [15]. Crystallographic data were collected on an Agilent Xcalibur Eos Gemini CCD plate diffractometer equipped with a graphite-monochromatic Cu-K*α* radiation (*λ* = 1.54178) Å at 293 (2) K. The data were corrected for absorption by using the program SADABS [16]. The structures were solved by direct methods with the SHELXTL software package [17]. All non-hydrogen atoms were refined anisotropically. The H atoms connected to C atoms were calculated theoretically, and those to O atoms were assigned by difference Fourier maps [18]. The absolute structure was determined by refinement of the Flack parameter [19], based on anomalous scattering. The structures were optimized by full-matrix least-squares techniques.

Crystal data of compound **1**: C_20_H_26_N_2_O_6_S_2_∙H_2_O, F.W. = 472.56, orthorhombic space group *P*2(1)2(1)2(1), unit cell dimensions *a* = 8.5746 (5) Å, *b* = 10.9602 (8) Å, *c* = 24.3158 (16) Å, *V* = 2285.2 (3) Å^3^, *α* = *β* = *γ* = 90°, *Z* = 4, *d*_calcd_ = 1.374 mg/m^3^, crystal dimensions 0.40 × 0.21× 0.13 mm, *μ* = 2.491 mm^−1^, *F*(000) = 1000. The 5006 measurements yielded 3321 independent reflections after equivalent data were averaged. The final refinement gave *R*_1_ = 0.0525 and *wR*_2_ = 0.1263 [*I* > 2*σ*(*I*)]. The absolute structure parameter was 0.01(3).

Crystal data of compound **2**: C_21_H_28_N_2_O_8_S_2_, F.W. = 500.57, monoclinic space group *P*2(1), unit cell dimensions *a* = 6.8423 (4) Å, *b* = 20.5136 (10) Å, *c* = 8.2231 (5) Å, *V* = 1130.48 (11) Å^3^, *α* = *γ* = 90°, *β* = 101.635 (2)°, *Z* = 2, *d*_calcd_ = 1.471 mg/m^3^, crystal dimensions 0.20 × 0.17× 0.10 mm, *μ* = 2.587 mm^−1^, *F*(000) = 528. The 6832 measurements yielded 2789 independent reflections after equivalent data were averaged. The final refinement gave *R*_1_ = 0.0514 and *wR*_2_ = 0.1199 [*I* > 2*σ*(*I*)]. The absolute structure parameter was 0.02(4).

Crystal data of compound **3**: C_20_H_20_N_2_O_7_S_2_∙CH_3_OH, F.W. = 496.54, orthorhombic space group *P*2(1)2(1)2(1), unit cell dimensions *a* = 10.1030 (6) Å, *b* = 10.8478 (5) Å, *c* = 19.6848 (10) Å, *V* = 2157.4 (2) Å^3^, *α* = *β* = *γ* = 90°, *Z* = 4, *d*_calcd_ = 1.529 mg/m^3^, crystal dimensions 0.25 × 0.17× 0.13 mm, *μ* = 2.711 mm^−1^, *F*(000) = 1040. The 4782 measurements yielded 3033 independent reflections after equivalent data were averaged. The final refinement gave *R*_1_ = 0.0510 and *wR*_2_ = 0.1001 [*I* > 2*σ*(*I*)]. The absolute structure parameter was 0.02(3).

### 3.6. Computational Section

Conformational searches were carried out via molecular mechanics with the MM+ method in HyperChem 8.0 software (Gainesville, FL, USA). Afterwards, the geometries were optimized at the gas-phase B3LYP/6-31G level in Gaussian09 software to afford the energy-minimized conformers. Then, the optimized conformers were subjected to the calculations of ECD spectra using the TD-DFT at BH&HLYP/TZVP level for **4** and PBE0/TZVP level for **5**. Simultaneously, solvent effects of the MeOH solution were evaluated at the same DFT level using the SCRF/PCM method [20].

### 3.7. Cytotoxic Assays

#### 3.7.1. Cell Culture

Liver cancer Huh7.5 cell line used was obtained from the American Type Culture Collection (ATCC). Human normal liver LO2 cell line used was purchased from China Center for Type Culture Collection (CCTCC). Huh7.5 cells and LO2 cells were cultured at 37 °C in RPMI-1640 medium and DMEM medium, respectively, supplemented with 10% fetal bovine serum (FBS, PAN Biotech, Aidenbach, Germany), 100 U/mL penicillin, and 100 mg/mL streptomycin. All experiments were carried out with the same batch of cell line between passages 2 and 5 [21].

#### 3.7.2. Cell Proliferation Assay

The cytotoxic activities of compounds **1**–**10** against Huh7.5 liver tumor cells and human normal liver LO2 cell line were determined by the 3-(4,5-dimethylthiazolyl-2)-2,5-diphenyltetrazolium bromide (MTT) assay. Briefly, 6 × 10^3^ of logarithmically growing Huh7.5 cells and human normal liver LO2 cell line were plated in the 96-well plate at 37 °C for 24 h. Then, cells were treated with DMSO (as the vehicle control) and increasing concentrations of test compounds (with the final concentration of 1, 2, 4, 5, 8, 10, 15, 20 μM) for 48 h, respectively. MTT solution (5 mg/mL, 20 μL per well) was added and incubated for another 4 h. After supernate from the wells were removed, DMSO was added to each well to dissolve purple crystals of formazan with gentle shaking for 10 min, and optical density at 490 nm was read by a multi-detection microplate reader (Infinite M1000 Pro, Tecan, Switzerland). Sorafenib was used as positive control. All the compounds and positive control were dissolved and diluted in DMSO. All tests were performed in triplicate. The values of relative cell viability were calculated as percentages of absorbance from the treated samples to absorbance from the vehicle control [21].

## 4. Conclusions

In conclusion, ten diketopiperazine alkaloids including four new derivatives, 5’-hydroxy-6’-ene-epicoccin G (**1**), 7-methoxy-7’-hydroxyepicoccin G (**2**), 8’-acetoxyepicoccin D (**3**), and 7’-demethoxyrostratin C (**4**), and a pair of new enantiomeric diketopiperazines (±)-5-hydroxydiphenylalazine A ((±)-**5**), along with five known analogues (**6**–**10**) were characterized from the deep sea-derived fungus *E. nigrum* SD-388. The discovery of these compounds might provide further insight into the biosynthesis of the diketopiperazine family and provide new targets for synthetic or biosynthetic studies. Compounds **4** and **10** exhibited potent cytotoxic activities against Huh7.5 liver cancer cells and may provide useful candidates for further study as antitumor agents.

## Figures and Tables

**Table 1:** ^1^H NMR spectroscopic data for compounds **1**–**5**^a^.

No.	1	2	3	4	5
2					4.17, t (6.4)
3	α 2.80, d (13.4)	α 2.75, d (13.5)	α 3.13, d (17.7)	α 2.68, m	a 3.02, dd (13.3, 6.4)
	β 2.28, m	β 2.35, dd (13.5, 8.5)	β 2.89, m	β 2.99, dd (14.8, 7.9)	b 2.95, dd (13.3, 6.4)
4	2.98, t (8.0)	2.92, t (8.5)	3.06, t (8.3)	3.18, t (7.9)	
6	α 2.22, m	α 2.54, dd (17.1, 4.7)	α 2.85, m	α 2.71, m	6.79, d (7.3)
	β 2.61, m	β 2.65, m	β 2.92, m	β 2.61, m	
7	α 2.19, m	3.82, ddd (10.1, 4.7, 2.0)	3.73, m	3.29, ddd (10.9, 4.7, 1.5)	6.96, m
	β 1.92, m				
8	4.36, m	4.56, m	4.01, dt (4.6, 2.4)	5.03, t (4.7)	6.67, td (7.3, 1.2)
9	4.33, m	4.33, m	4.67, dd (8.3, 2.4)	4.41, dd (7.9, 4.7)	6.98, m
3’	α 2.42, dd (12.5,4.9)	α 2.73, d (13.4)	α 2.93, m	α 2.74, m	6.83, s
	β 2.26, m	β 2.34, dd (13.4, 8.5)	β 3.29, d (19.4)	β 2.93, dd (14.8, 8.0)	
4’	2.09, m	2.91, t (8.5)	3.17, d (8.1)	3.23, t (8.0)	
5’	4.11, m				7.27, d (7.3)
6’	5.68, d (9.8)	α 2.40, dd (16.8, 4.4)	α 2.89, m	α 2.66, m	7.40, t (7.3)
		β 2.61, m	β 2.97, m	β 2.27, dt (16.7, 4.1)	
7’	5.53, d (9.8)	4.12, ddd (10.2, 4.4, 2.0)	3.92, m	α 1.62, td (12.5, 5.0)	7.32, t (7.3)
				β 1.89, m	
8’	4.08, m	4.29, s	5.04, dd (4.3, 2.0)	4.81, m	7.40, t (7.3)
9’	3.41, dd (12.0, 8.1)	4.34, m	4.83, dd (8.1, 2.0)	4.36, m	7.27, d (7.3)
1-NMe					2.69, s
1’-NH					-^b^
2-SMe	1.95, s	1.93, s			
2’-SMe	2.08, s	1.92, s			
2″			2.04, s		
5-OH					8.50, br s
8-OH	5.36, d (3.2)	5.44, br s	6.25, d (2.4)	5.64, d (4.7)	
5’-OH	5.92, s				
7’-OH		5.19, br s			
8’-OH	5.28, d (5.8)	5.44, br s		5.49, d (4.0)	
7-OMe		3.25, s		3.21, s	

*^a^* Data collected at 500 MHz in DMSO-*d*_6_. *^b^* Data not detected.

**Table 2:** ^13^C NMR spectroscopic data for compounds **1**–**5**^a^.

No.	1	2	3	4	5
1	168.7, C	165.5, C	158.3, C	162.2, C	167.2, C
2	71.4, C	71.6, C	71.1, C	76.2, C	55.6, CH
3	34.3, CH_2_	34.4, CH_2_	41.4, CH_2_	32.6, CH_2_	34.8, CH_2_
4	44.0, CH	43.8, CH	45.0, CH	46.4, CH	122.0, C
5	207.5, C	206.6, C	207.1, C	207.8, C	156.1, C
6	33.8, CH_2_	40.4, CH_2_	43.2, CH_2_	40.7, CH_2_	115.1, CH
7	25.9, CH_2_	75.8, CH	41.3, CH	75.5, CH	127.8, CH
8	63.6, CH	61.5, CH *^b^*	65.2, CH	61.9, CH	118.4, CH
9	64.8, CH	63.2, CH	60.3, CH	63.2, CH	131.2, CH
1’	165.5, C	165.4, C	158.9, C	162.2, C	162.4, C
2’	72.9, C	71.6, C	71.7, C	76.4, C	132.2, C
3’	35.1, CH_2_	34.1, CH_2_	41.5, CH_2_	32.2, CH_2_	117.7, CH
4’	43.4, CH	43.5, CH	45.3, CH	46.7, CH	133.8, C
5’	71.3, CH	207.4, C	206.3, C	208.6, C	129.4, CH
6’	133.3, CH	43.3, CH_2_	43.5, CH_2_	33.9, CH_2_	128.1, CH
7’	129.9, CH	65.7, CH	38.8, CH	25.4, CH_2_	127.9, CH
8’	68.9, CH	68.1, CH *^b^*	67.4, CH	60.8, CH	128.1, CH
9’	67.8, CH	62.7, CH	57.5, CH	65.8, CH	129.4, CH
1″			168.8, C		
2″			20.6, CH_3_		
1-NMe					34.5, CH_3_
2-SMe	14.4, CH_3_	14.2, CH_3_			
2’-SMe	14.2, CH_3_	13.9, CH_3_			
7-OMe		55.8, CH_3_		56.0, CH_3_	

*^a^* Data collected at 125 MHz in DMSO-*d*_6_. *^b^* Assigned by HSQC experiment.

## Supplementary Materials

Selected 1D and 2D NMR, HRESIMS, and ECD spectra of compounds **1**–**5** are available online at: https://www.mdpi.com/1660-3397/18/3/160/s1.
