# Supplementary material for: Cytotoxic Thiodiketopiperazine Derivatives from the Deep Sea-Derived Fungus Epicoccum nigrum SD-388
Source: Mar Drugs. 2020 Mar 13;18(3):160. doi: 10.3390/md18030160 (PMC7143119; doi:10.3390/md18030160)

Supplementary Material

Antitumor thiodiketopiperazine derivatives from the deep sea-derived fungus *Epicoccum nigrum* SD-388

Lu-Ping Chi [1,2,3], Xiao-Ming Li [1,2], Li Li [1,2,3], Xin Li [1,2,*] and Bin-Gui Wang [1,2,4,*]

Content

Figure S1. ^{1}H NMR (500 MHz, DMSO-d_6) spectrum of compound **1**;

Figure S2. ^{13}C NMR (125 MHz, DMSO-d_6) and DEPT spectra of compound **1**;

Figure S3. COSY spectrum of compound **1**;

Figure S4. HMBC spectrum of compound **1**;

Figure S5. NOESY spectrum of compound **1**;

Figure S6. HR-ESI-MS spectrum of compound **1**;

Figure S7. Crystal packing of compound **1** at 293(2) K;

Figure S8. ^{1}H NMR (500 MHz, DMSO-d_6) spectrum of compound **2**;

Figure S9. ^{13}C NMR (125 MHz, DMSO-d_6) and DEPT spectra of compound **2**;

Figure S10. COSY spectrum of compound **2**;

Figure S11. HSQC spectrum of compound **2**;

Figure S12. HMBC spectrum of compound **2**;

Figure S13. NOESY spectrum of compound **2**;

Figure S14. HR-ESI-MS spectrum of compound **2**;

Figure S15. Crystal packing of compound **2** at 293(2) K;

Figure S16. ^{1}H NMR (500 MHz, DMSO-d_6) spectrum of compound **3**;

Figure S17. ^{13}C NMR (125 MHz, DMSO-d_6) and DEPT spectra of compound **3**;

Figure S18. COSY spectrum of compound **3**;

Figure S19. HMBC spectrum of compound **3**;

Figure S20. NOESY spectrum of compound **3**;

Figure S21. HR-ESI-MS spectrum of compound **3**;

Figure S22. Crystal packing of compound **3** at 293(2) K;

Figure S23. ^{1}H NMR (500 MHz, DMSO-d_6) spectrum of compound **4**;

Figure S24. ^{13}C NMR (125 MHz, DMSO-d_6) and DEPT spectra of compound **4**;

Figure S25. COSY spectrum of compound **4**;

Figure S26. HSQC spectrum of compound **4**;

Figure S27. HMBC spectrum of compound **4**;

Figure S28. NOESY spectrum of compound **4**;

Figure S29. HR-ESI-MS spectrum of compound **4**;

Figure S30. ECD spectrum of compound **4**;

Figure S31. ^{1}H NMR (500 MHz, DMSO-d_6) spectrum of compound **5**;

Figure S32. ^{13}C NMR (125 MHz, DMSO-d_6) and DEPT spectra of compound **5**;

Figure S33. COSY spectrum of compound (±)-**5**;

Figure S34. HMBC spectrum of compound (±)-**5**;

Figure S35. HR-ESI-MS spectrum of compound (±)-**5**;

Figure S36. ECD spectrum of compound (±)-**5**;

Figure S37. Chiral HPLC profile of compound (±)-**5**;

Figure S38. ECD spectrum of compound (+)-**5**;

Figure S39. ECD spectrum of compound (–)-**5**.

Figure S1. ^{1}H NMR (500 MHz, DMSO-d_6) spectrum of compound **1**.

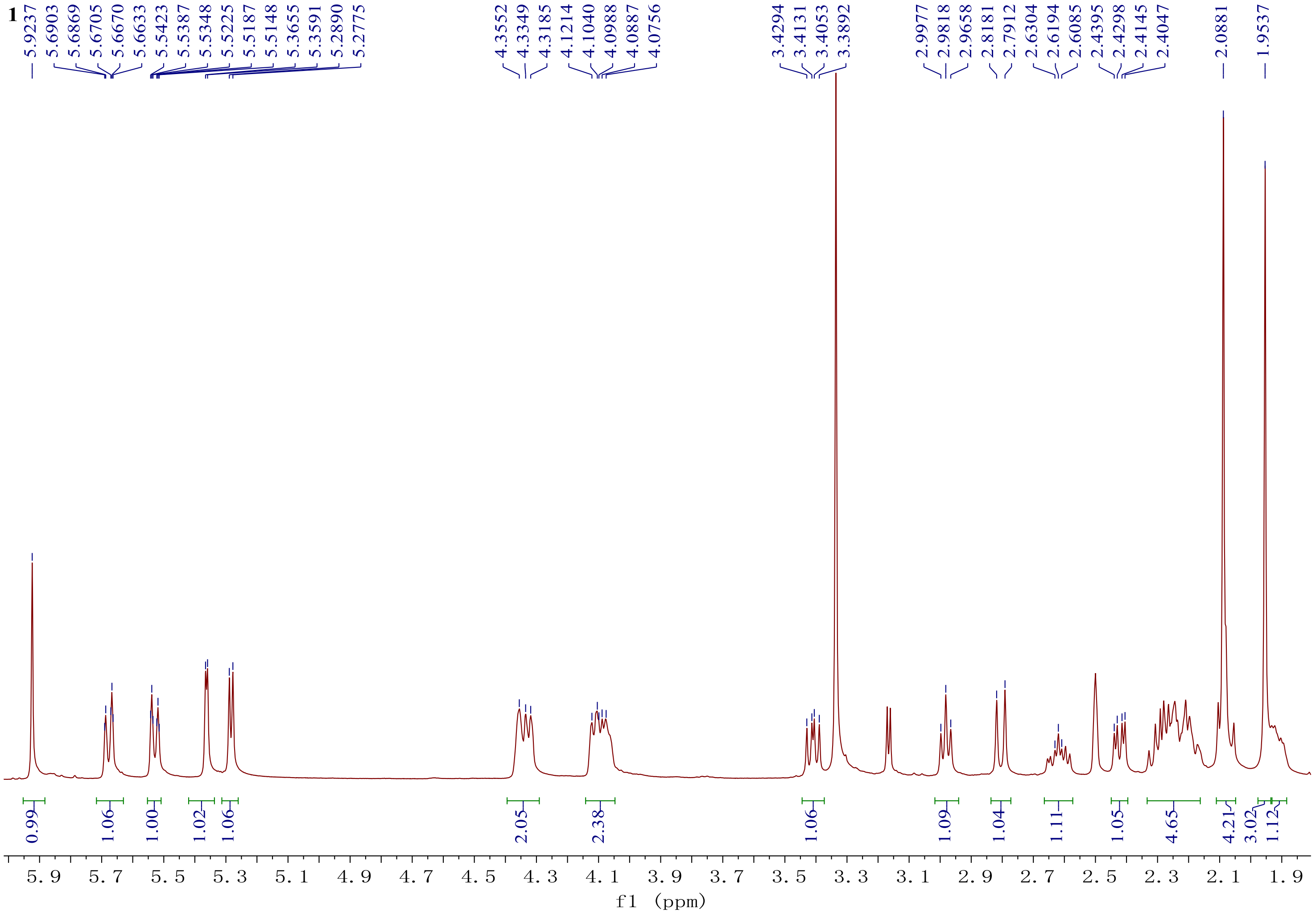

Figure S2. ^{13}C NMR and DEPT (125 MHz, DMSO-d_6) spectrum of compound **1**.

1

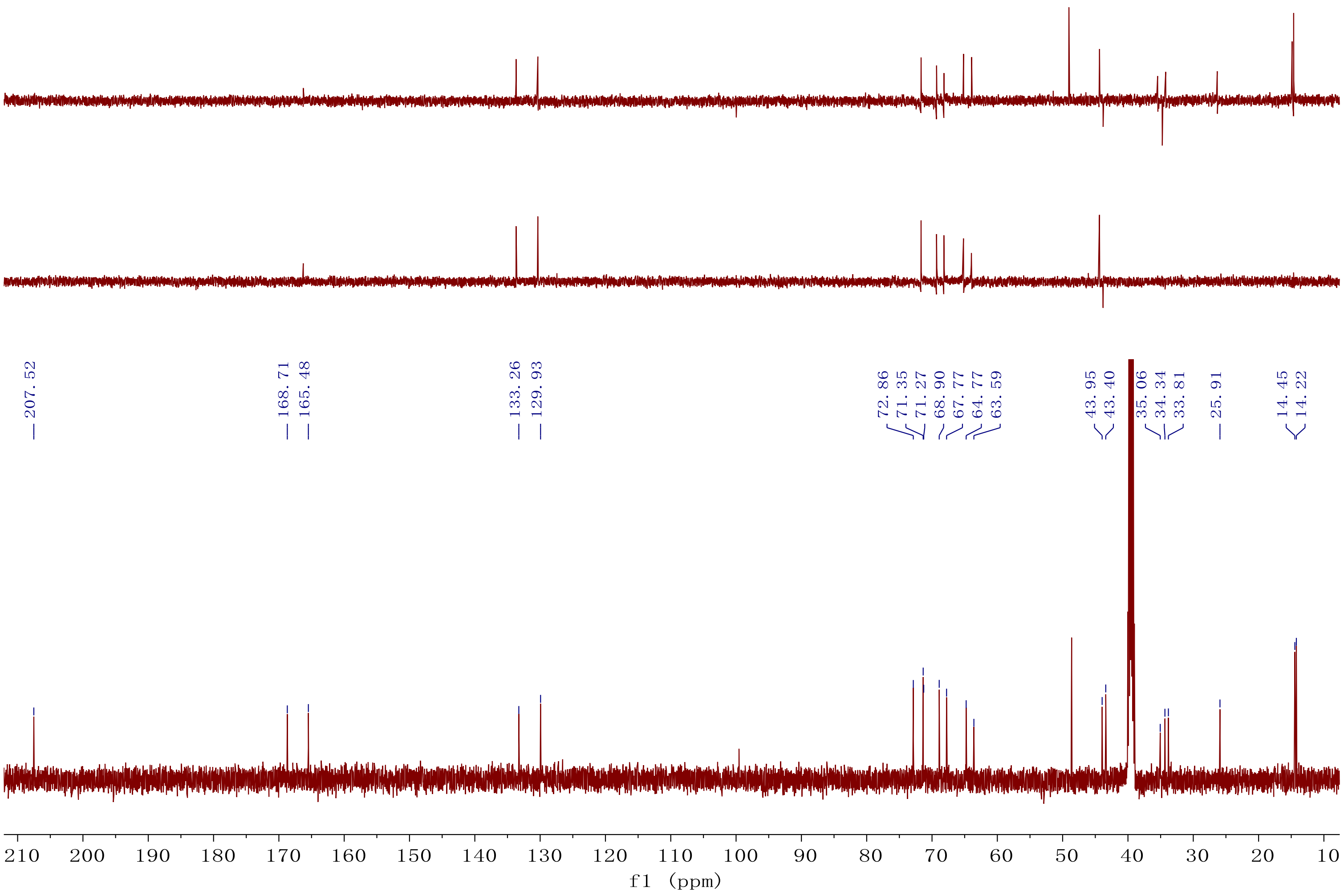

Figure S3. COSY spectrum of compound **1**.

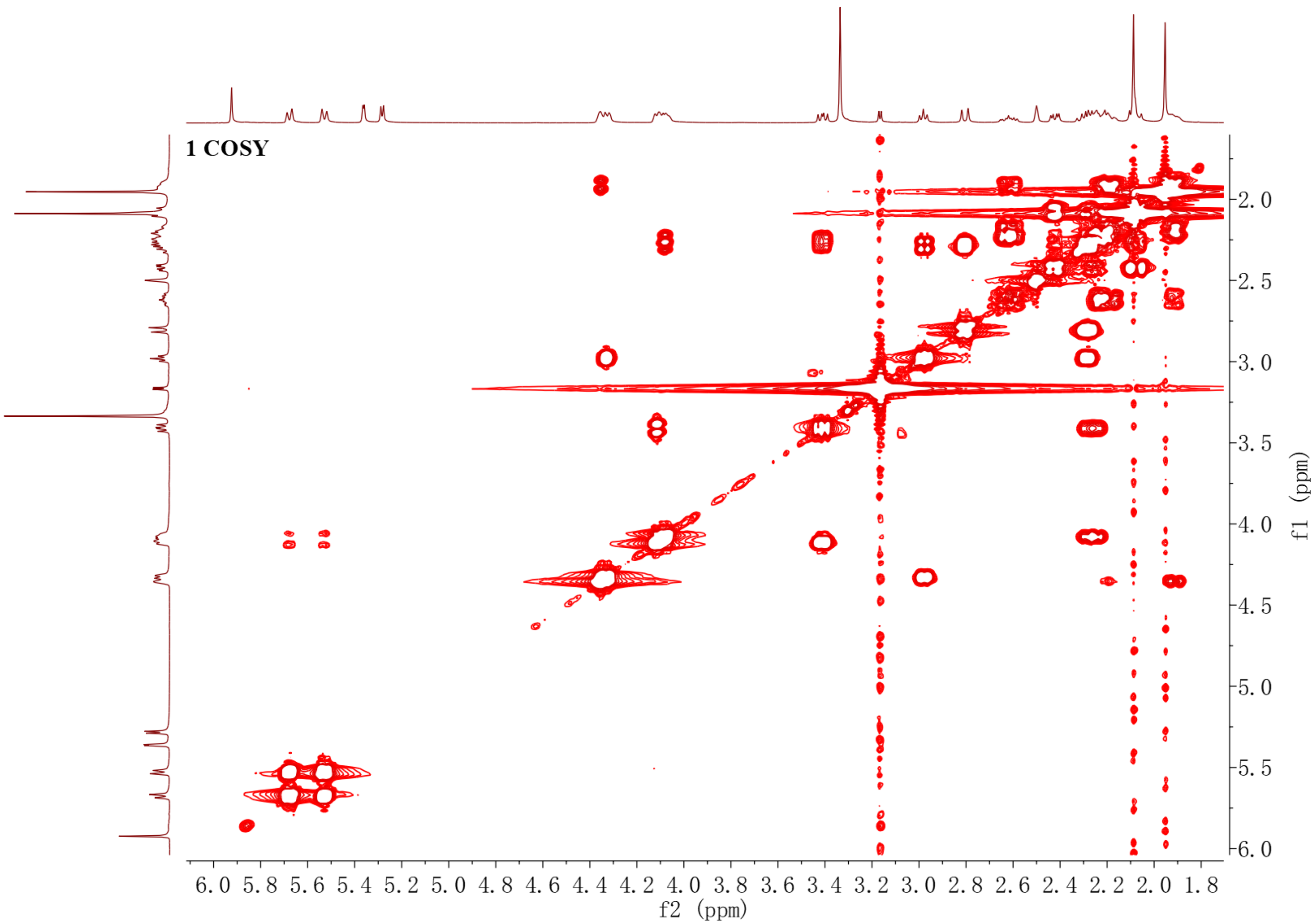

Figure S4. HMBC spectrum of compound **1**.

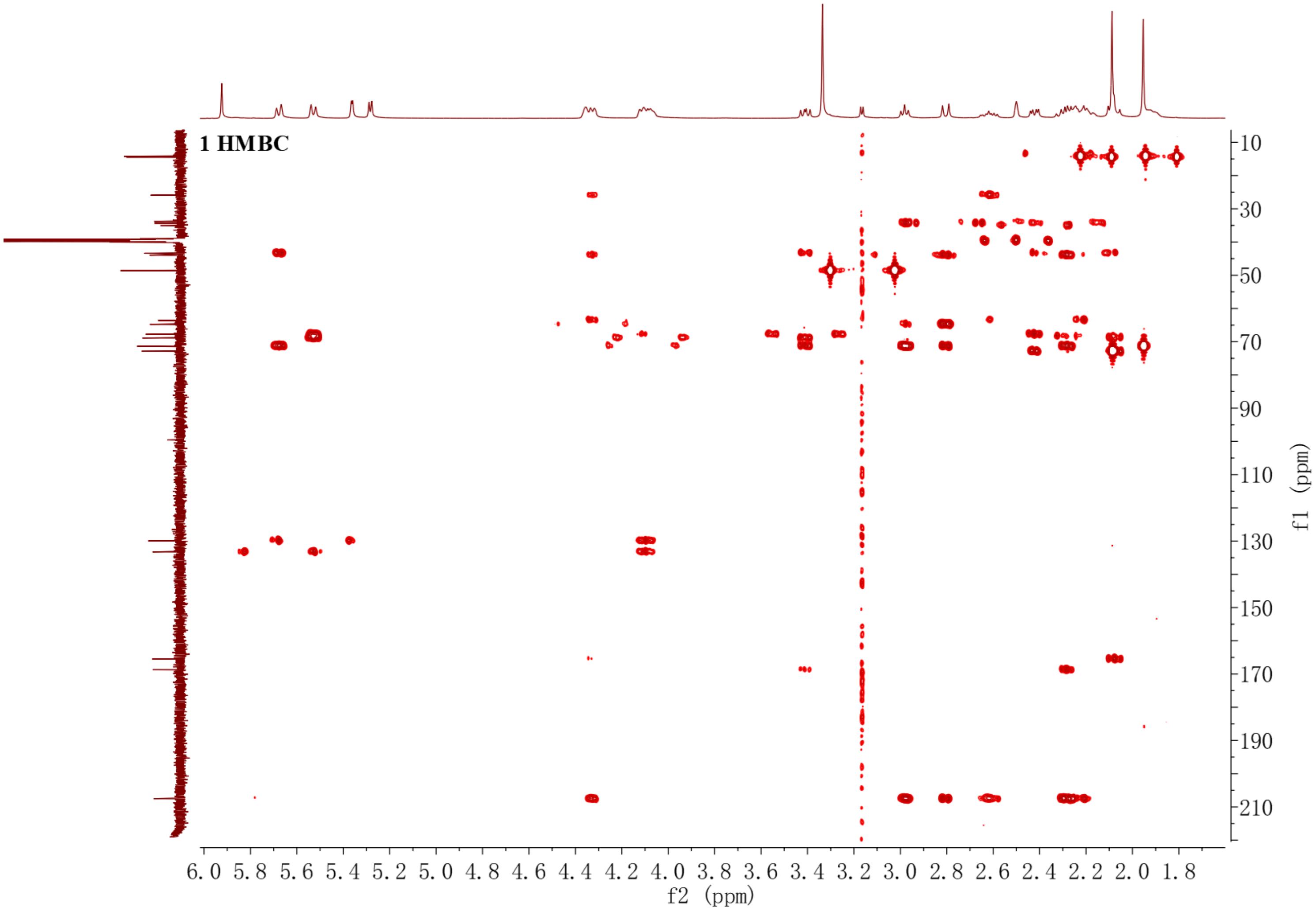

Figure S5. NOESY spectrum of compound **1**.

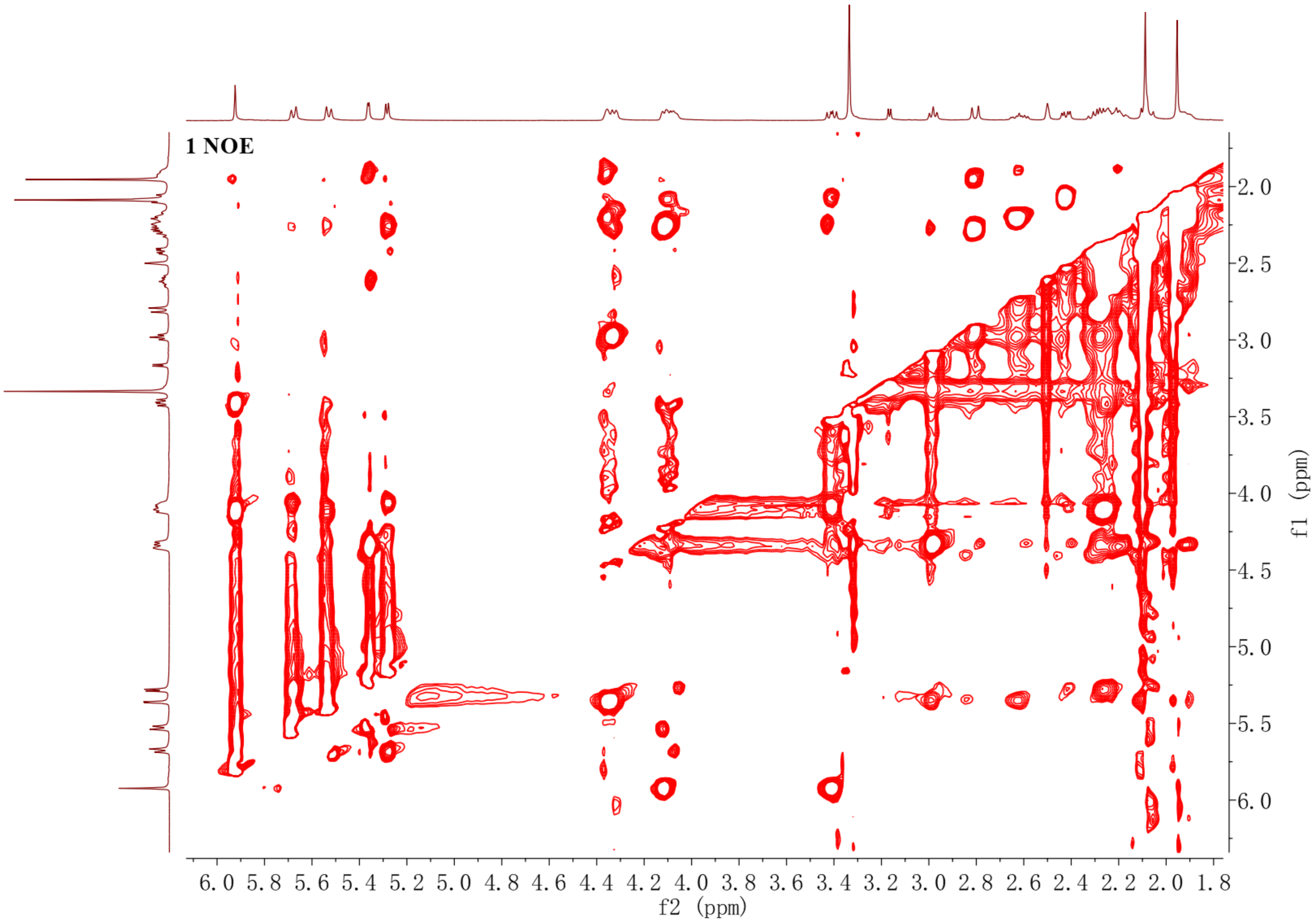

Figure S6. HR-ESI-MS of compound **1**.

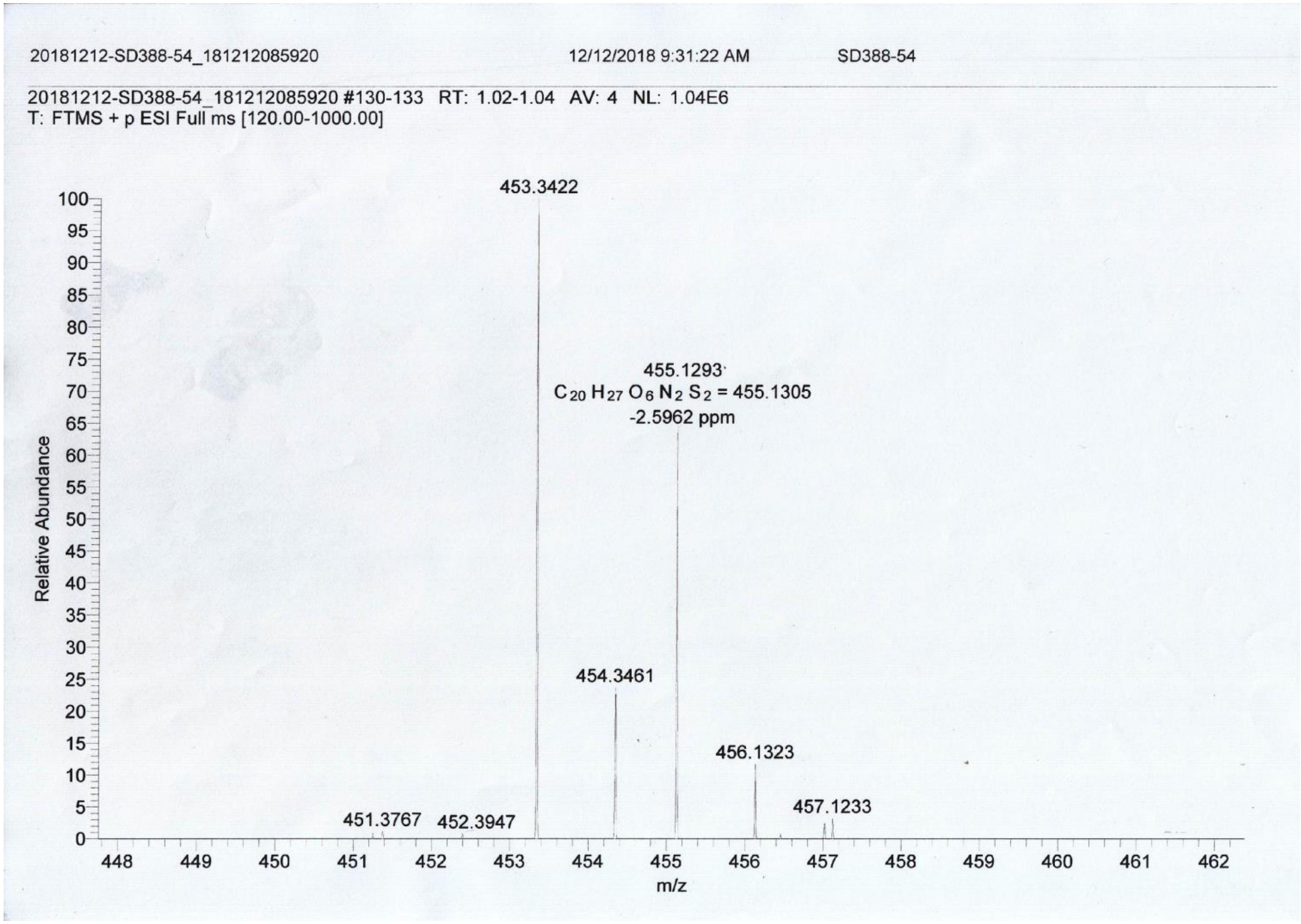

Figure S7. Crystal packing of compound **1** at 293(2) K;

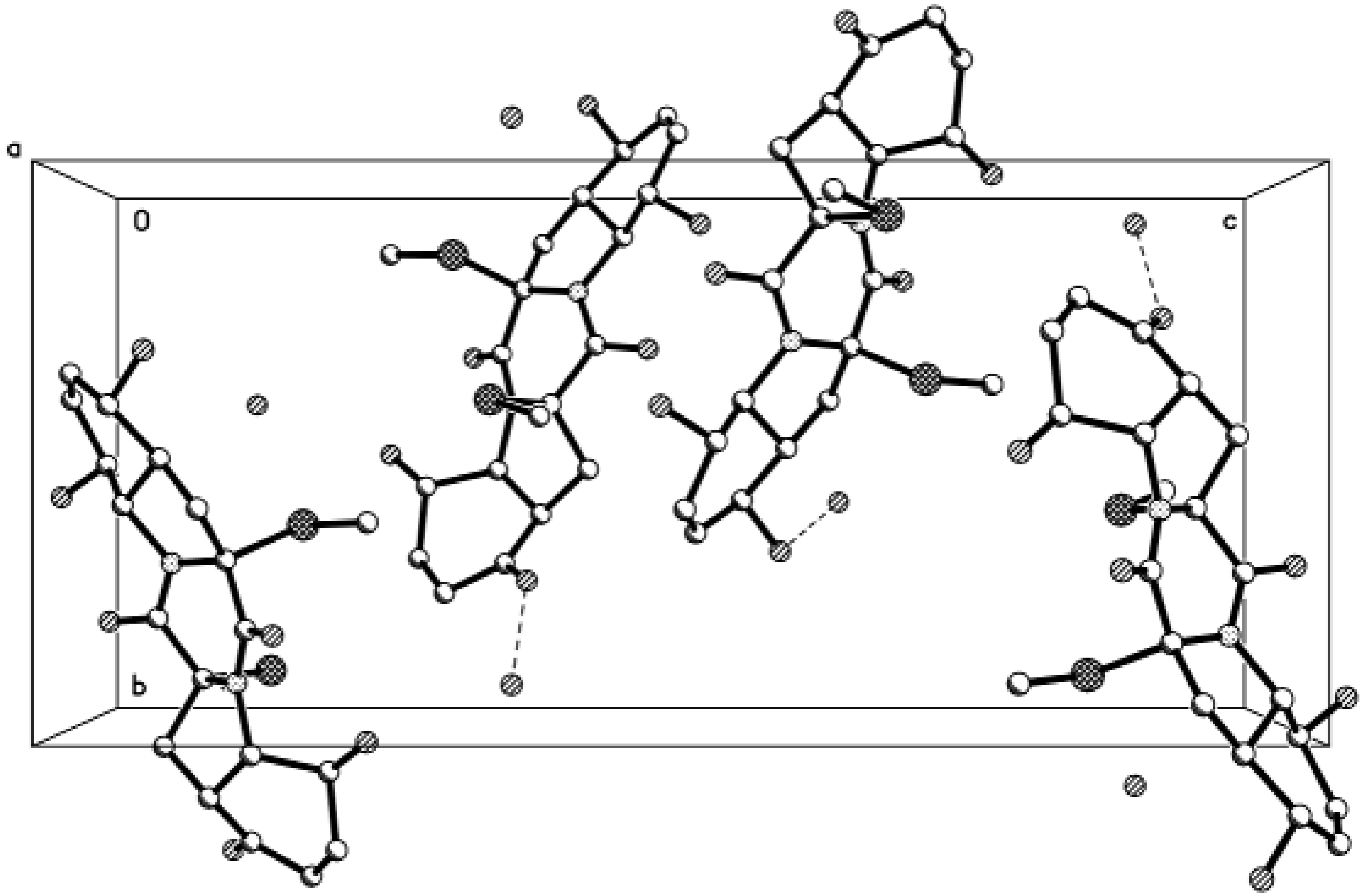

Figure S8. ^{1}H NMR (500 MHz, DMSO-d_6) spectrum of compound **2**.

2

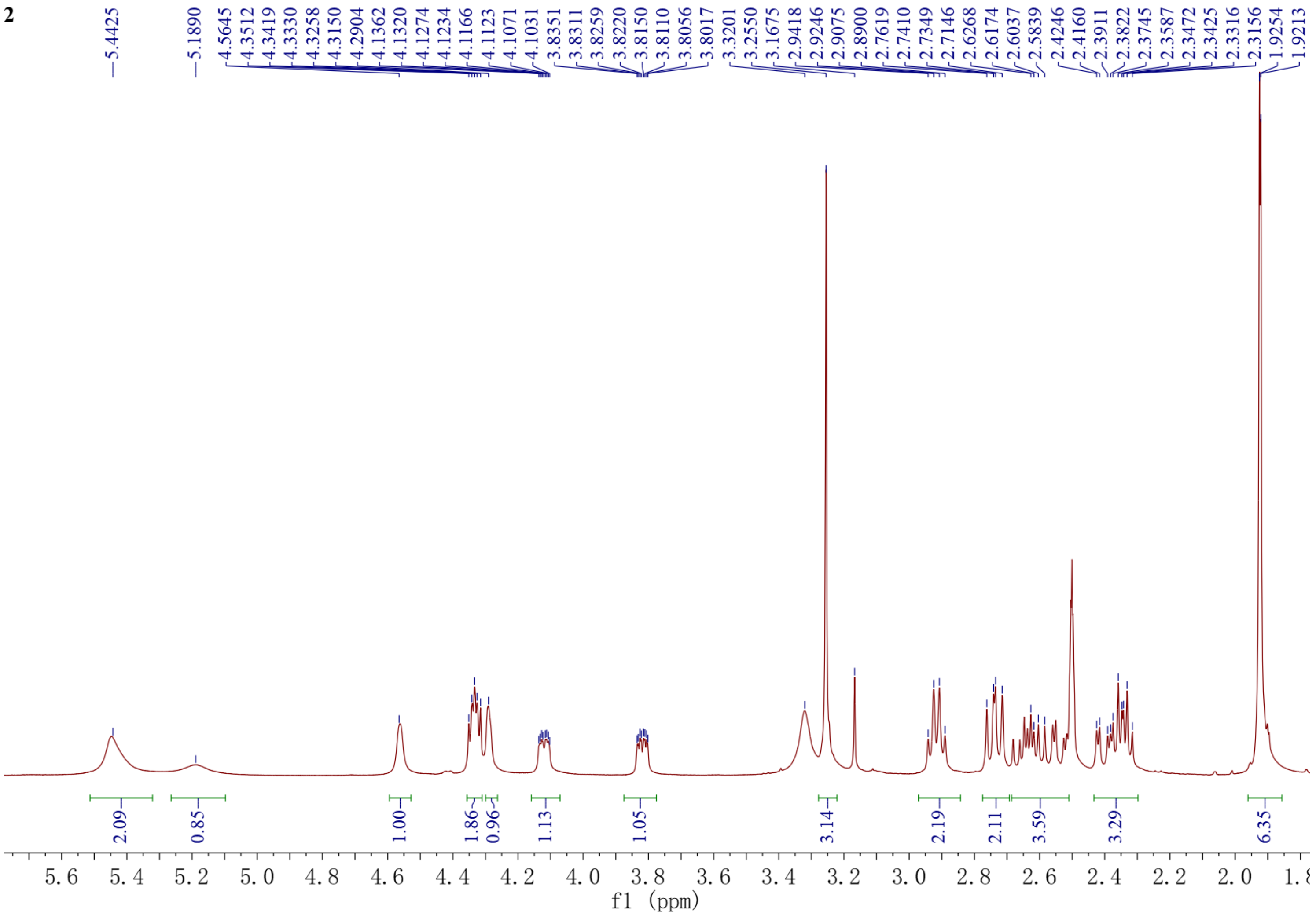

Figure S9. ^{13}C NMR and DEPT (125 MHz, DMSO-d_6) spectrum of compound **2**.

2

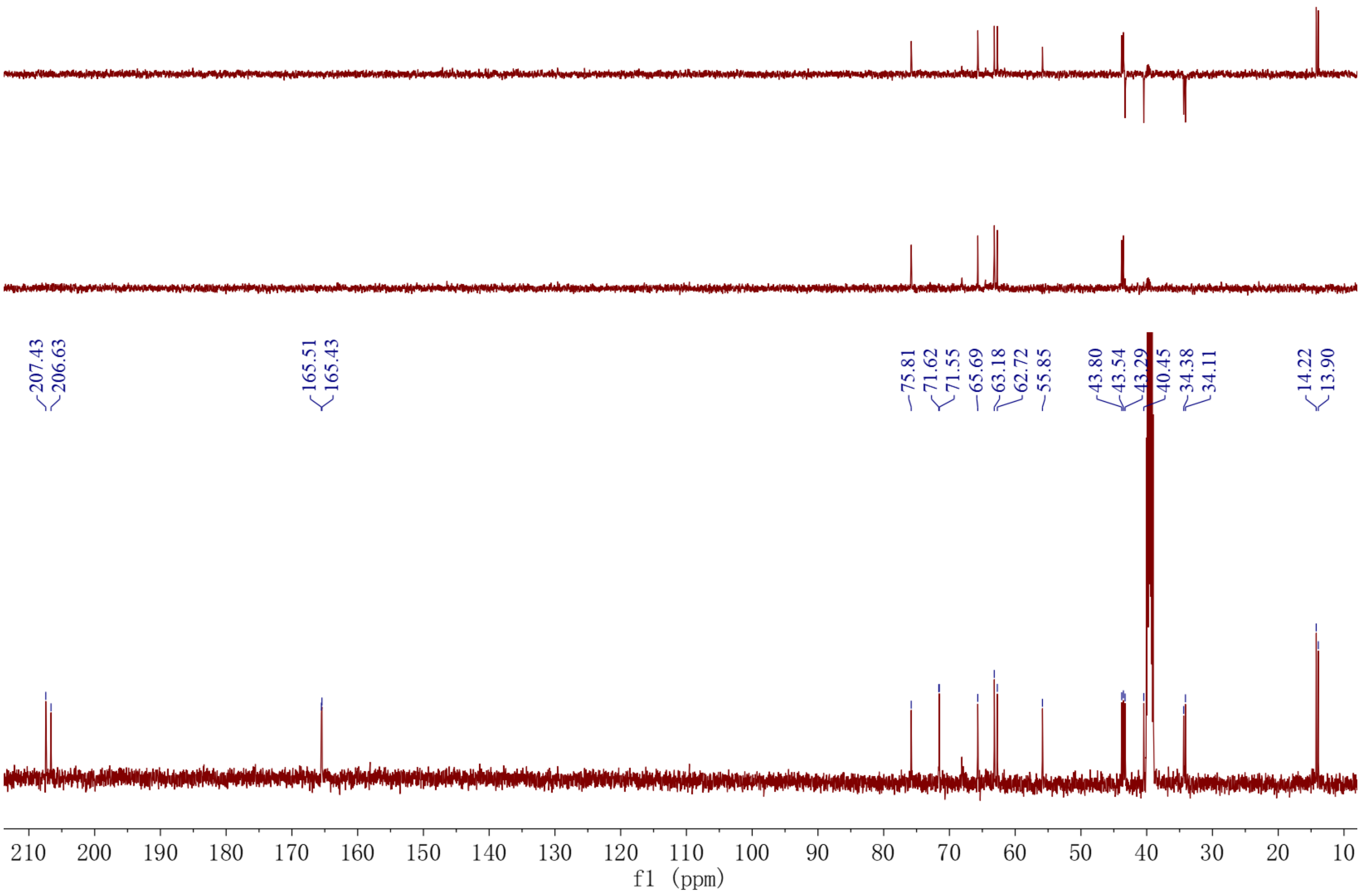

Figure S10. COSY spectrum of compound **2**.

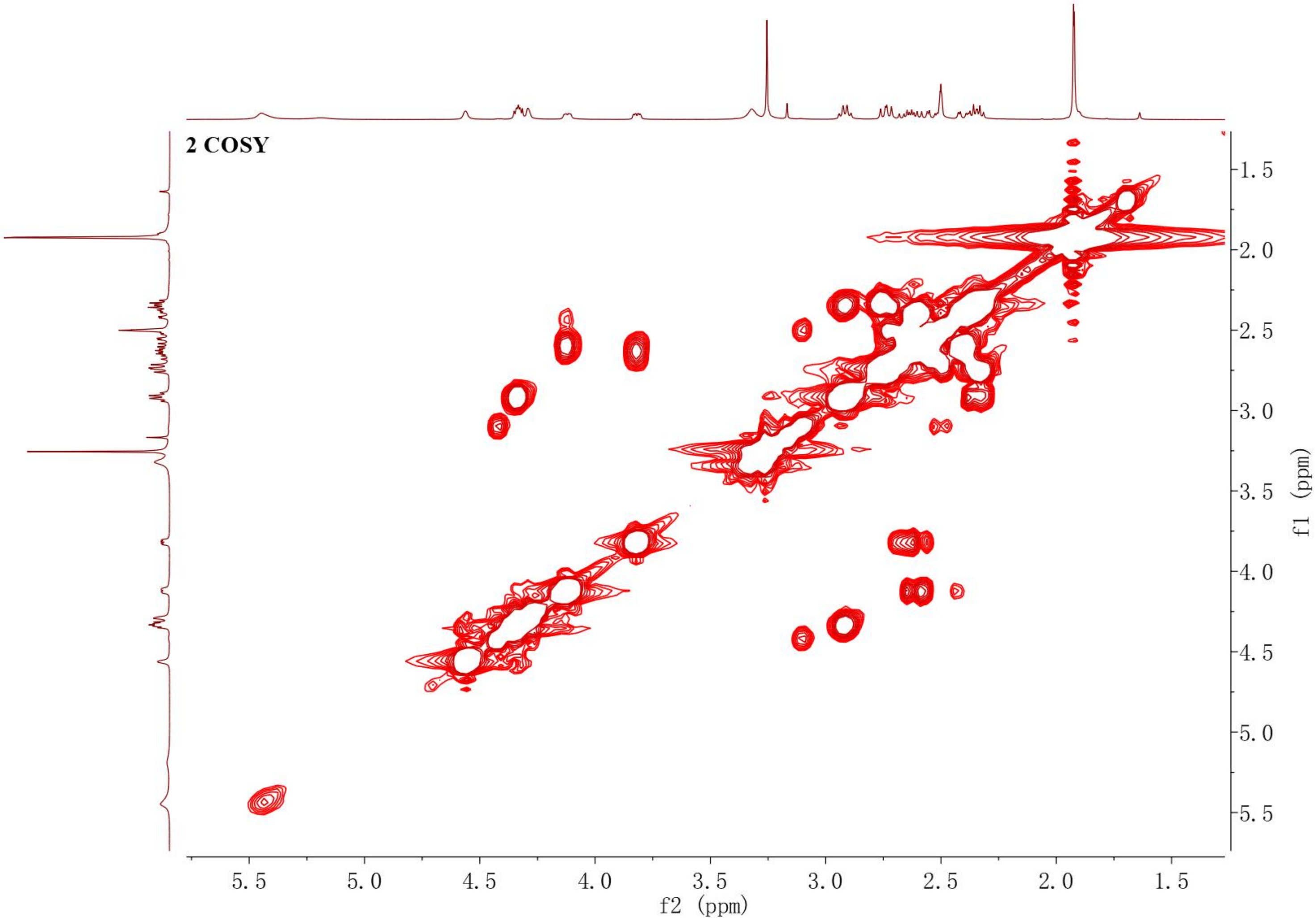

Figure S11. HSQC spectrum of compound **2**.

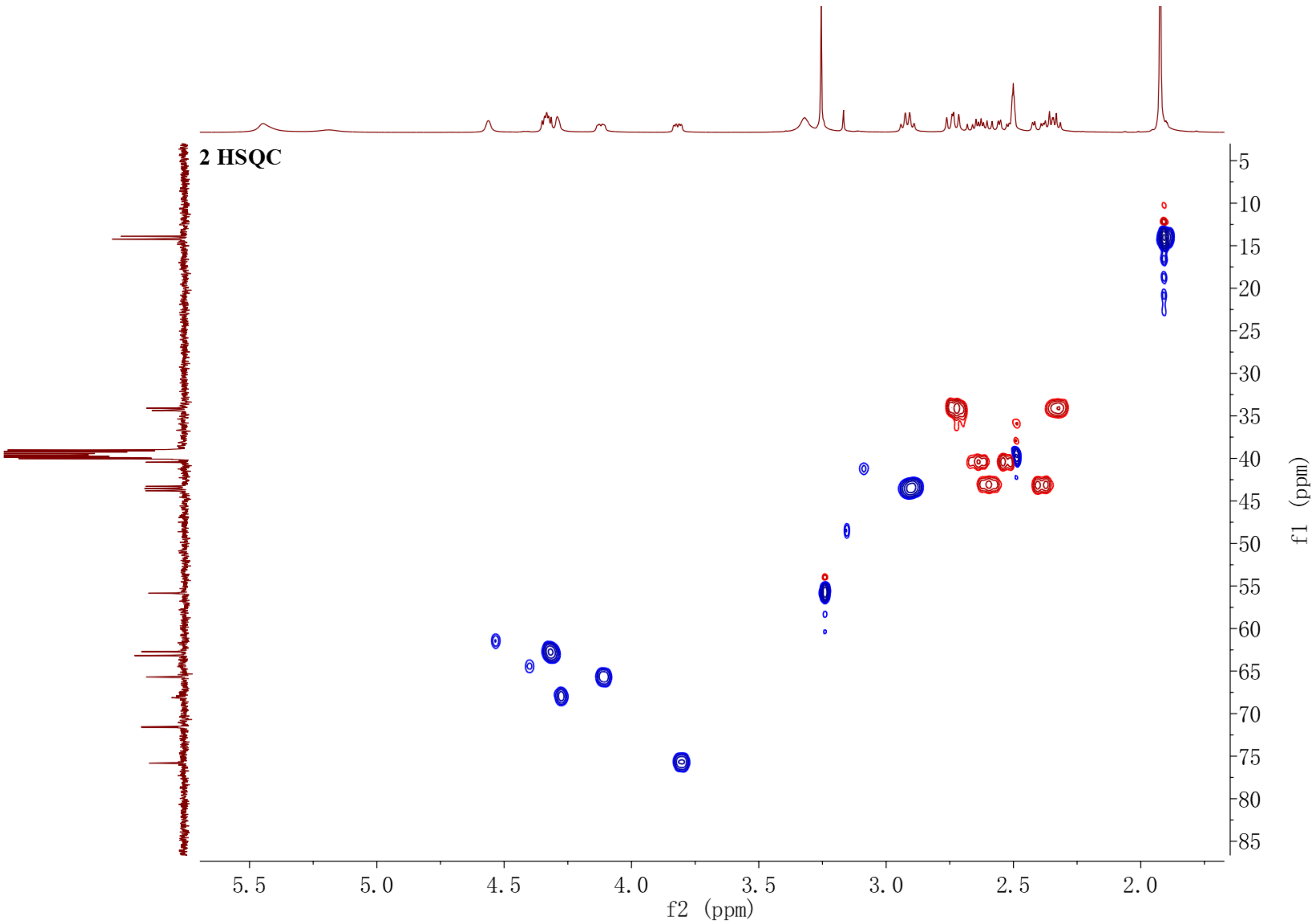

Figure S12. HMBC spectrum of compound **2**.

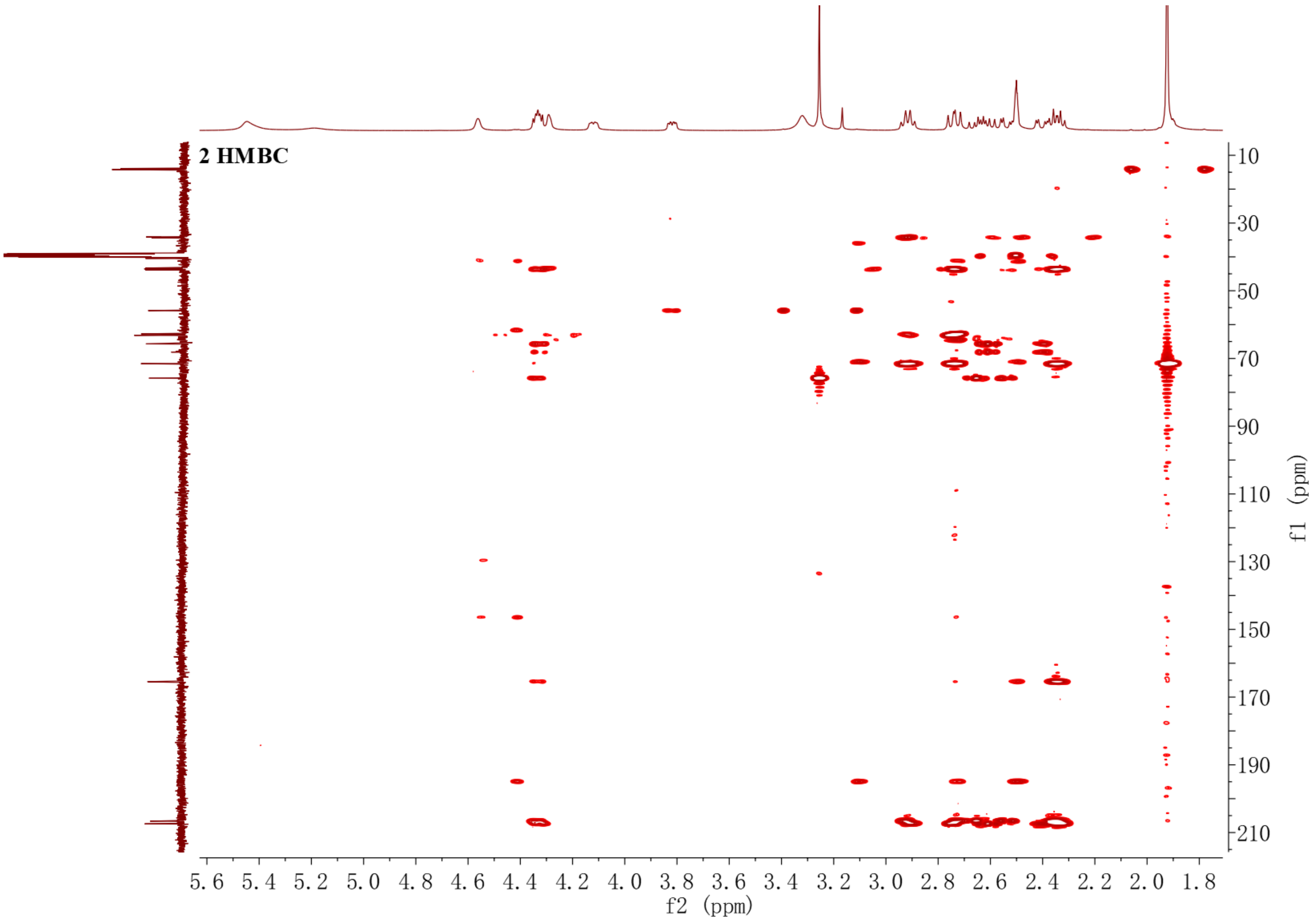

Figure S13. NOESY spectrum of compound **2**.

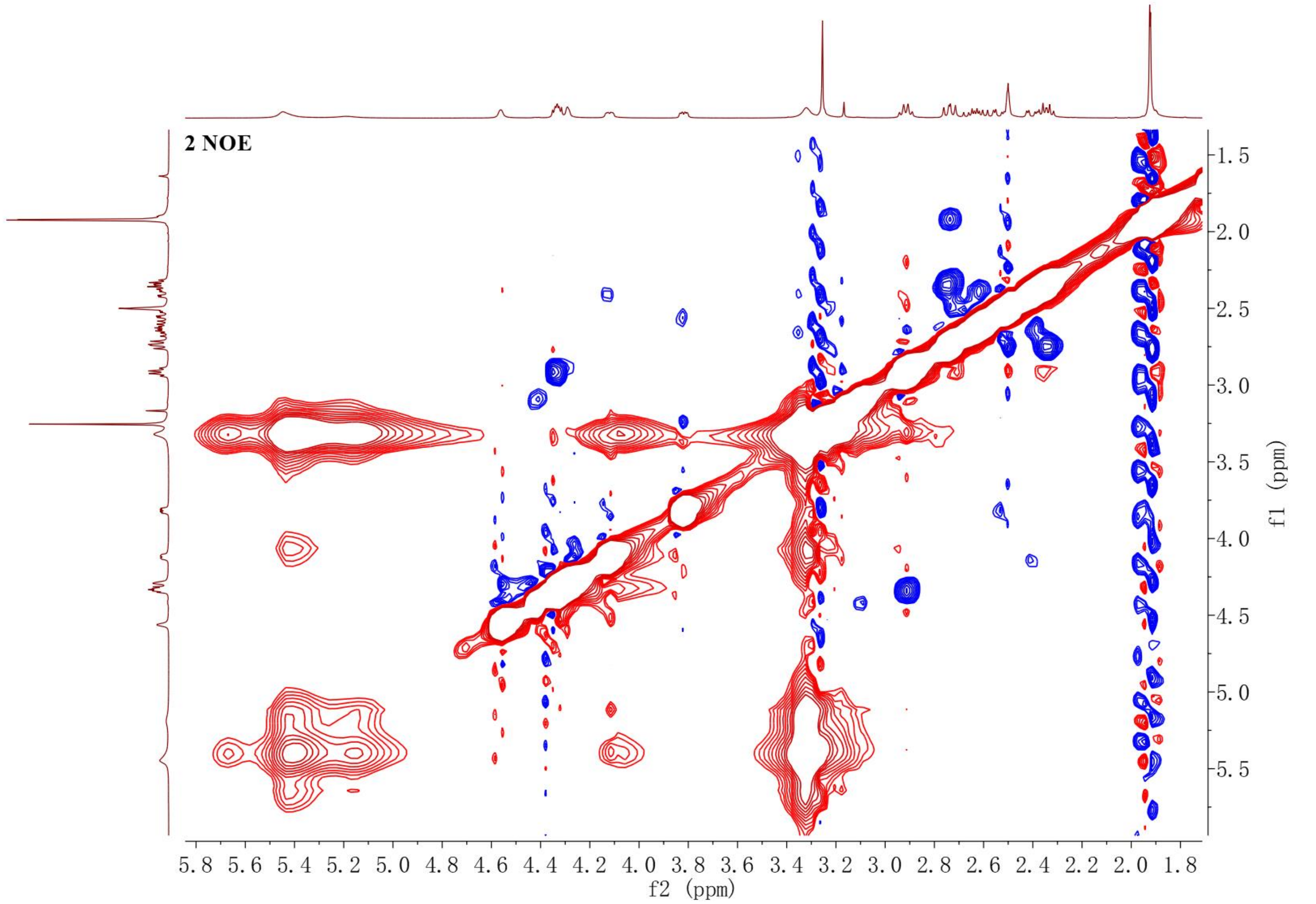

Figure S14. HR-ESI-MS of compound **2**.

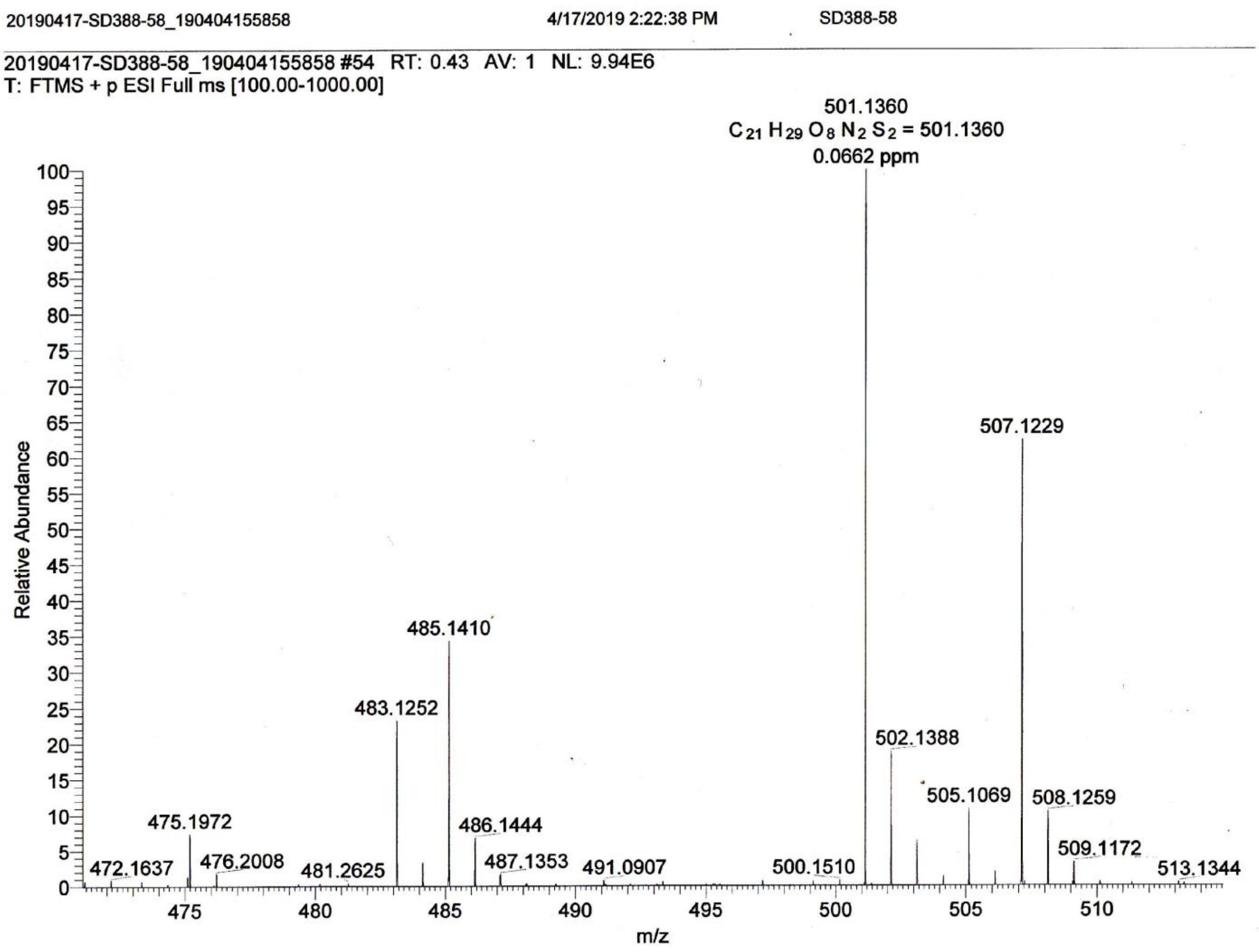

Figure S15. Crystal packing of compound **2** at 293(2) K;

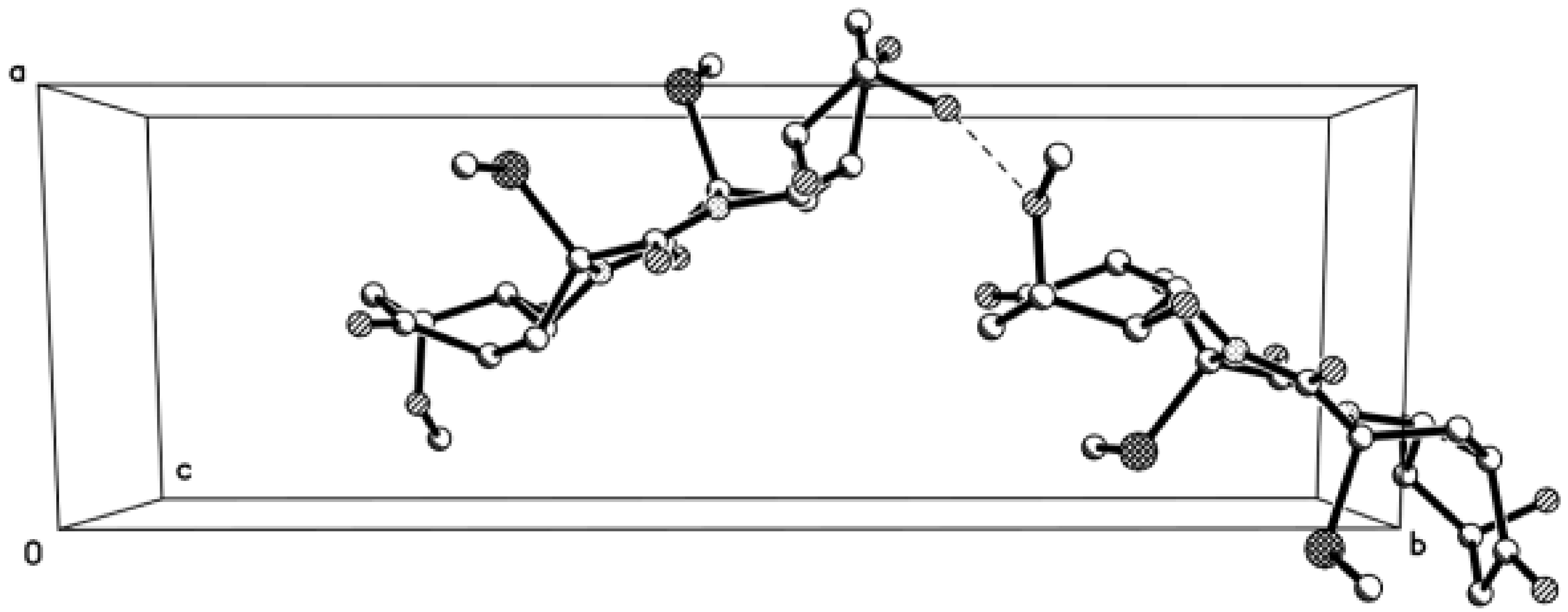

Figure S16. ^{1}H NMR (500 MHz, DMSO-d_6) spectrum of compound **3**.

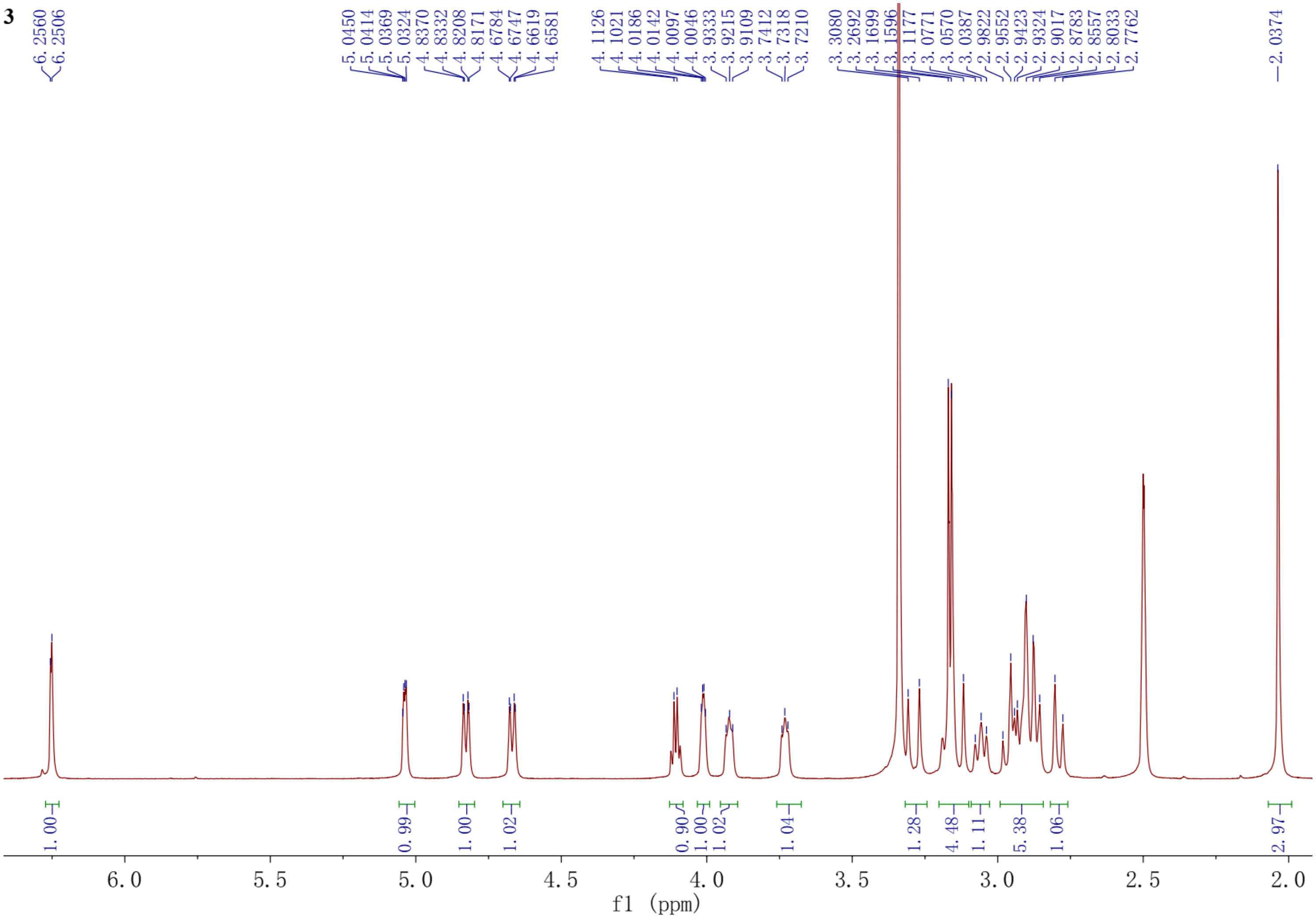

Figure S17. ^{13}C NMR and DEPT (125 MHz, DMSO-d_6) spectrum of compound **3**.

3

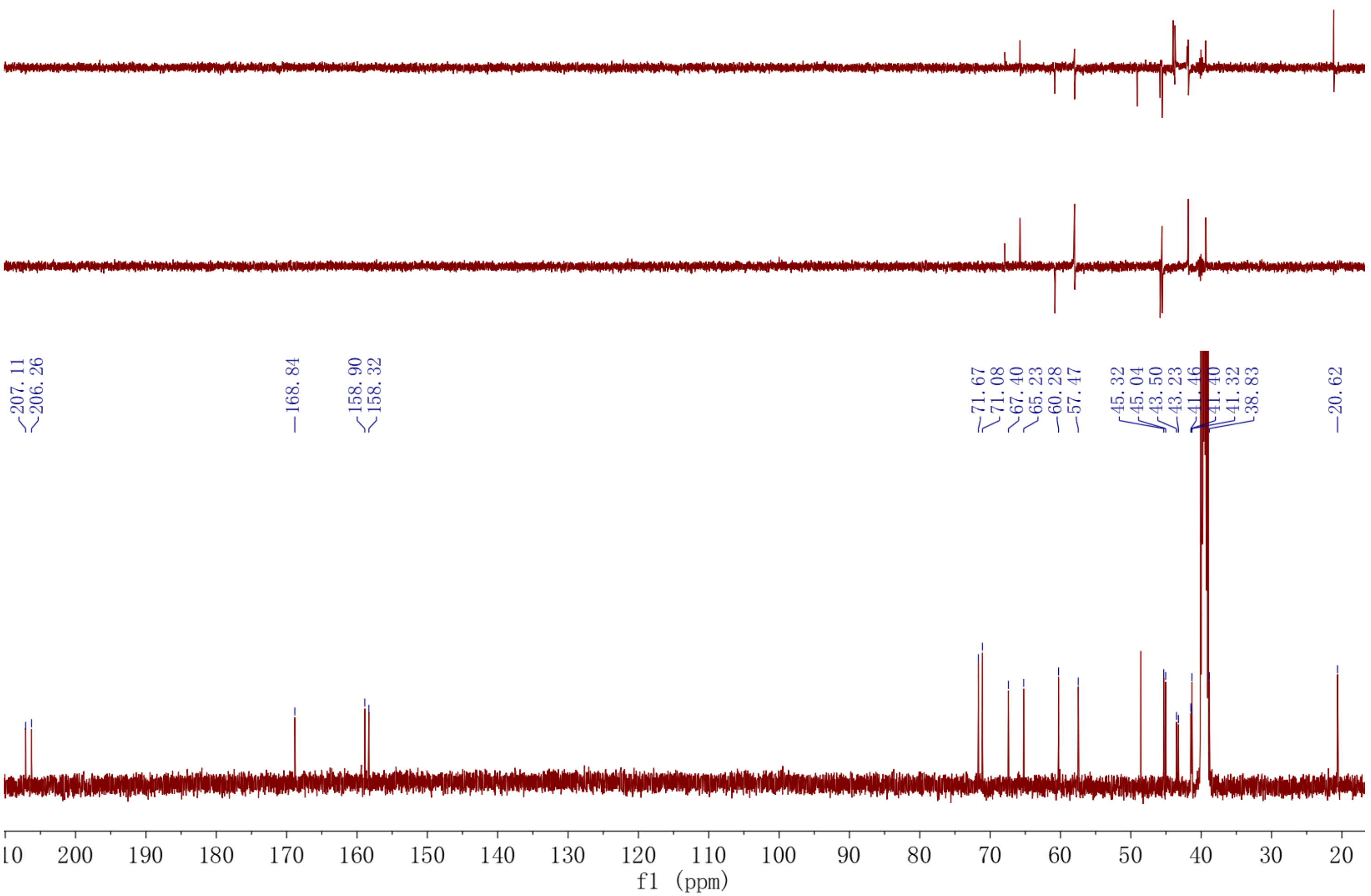

Figure S18. COSY spectrum of compound **3**.

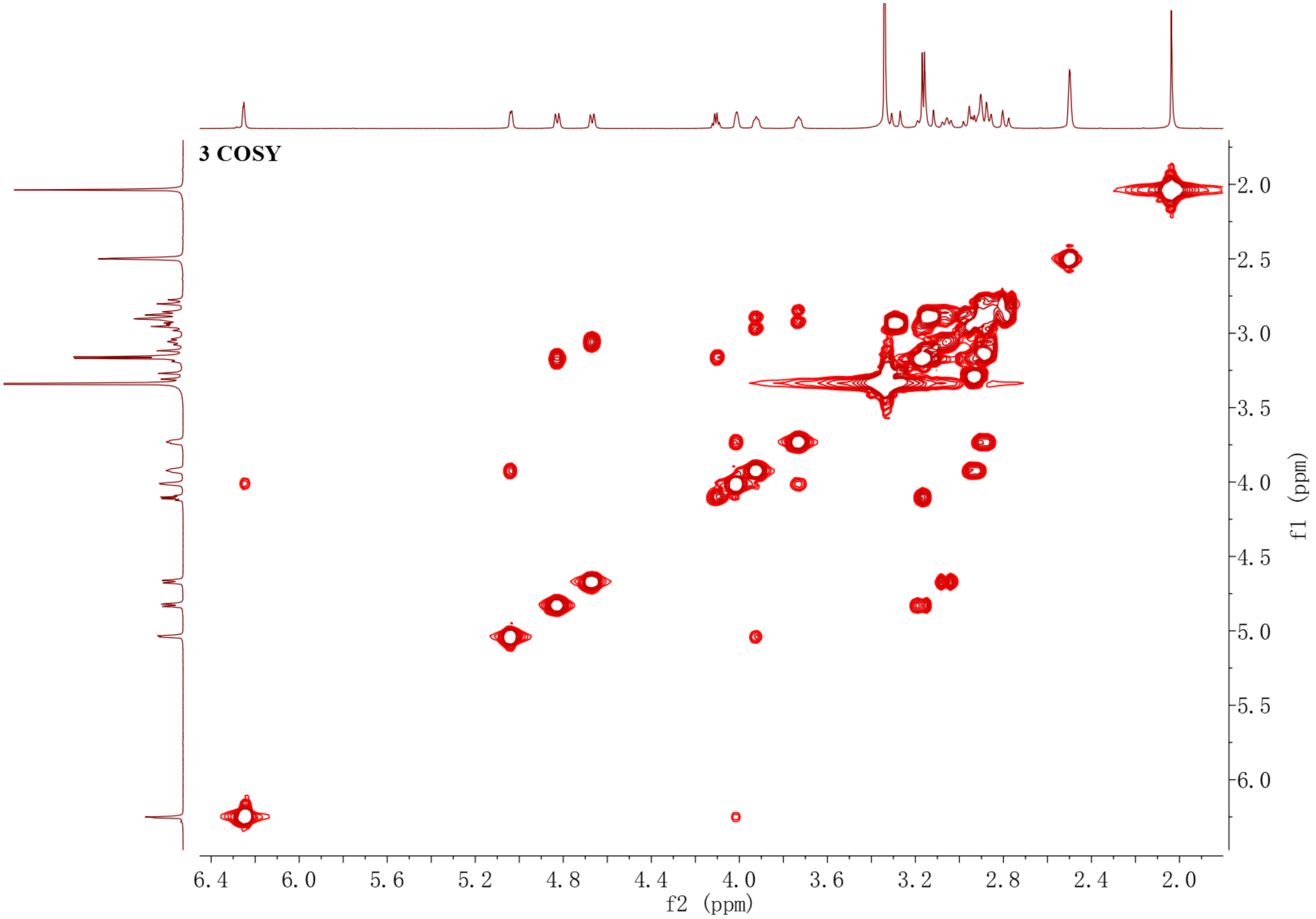

Figure S19. HMBC spectrum of compound **3**.

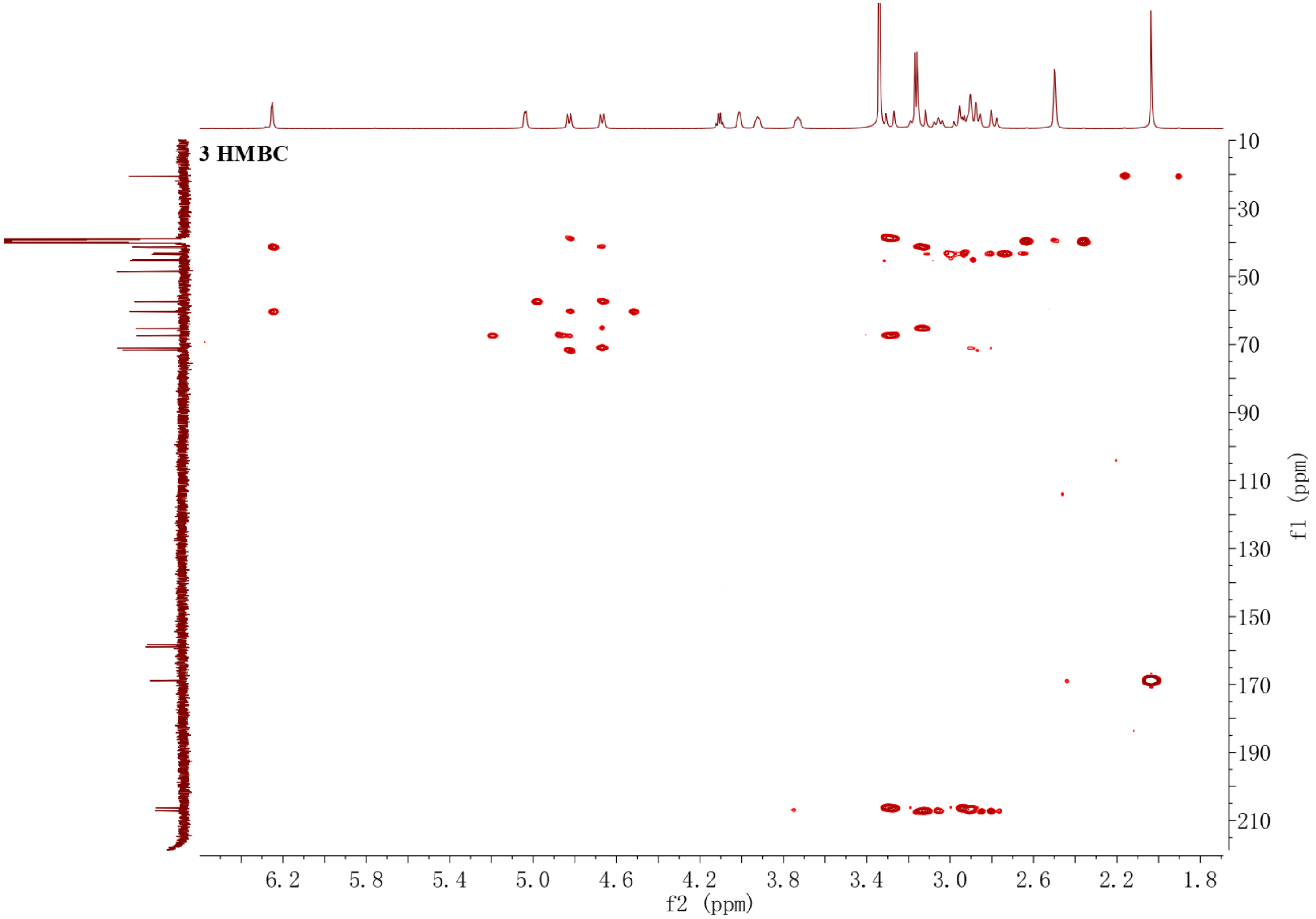

Figure S20. NOESY spectrum of compound **3**.

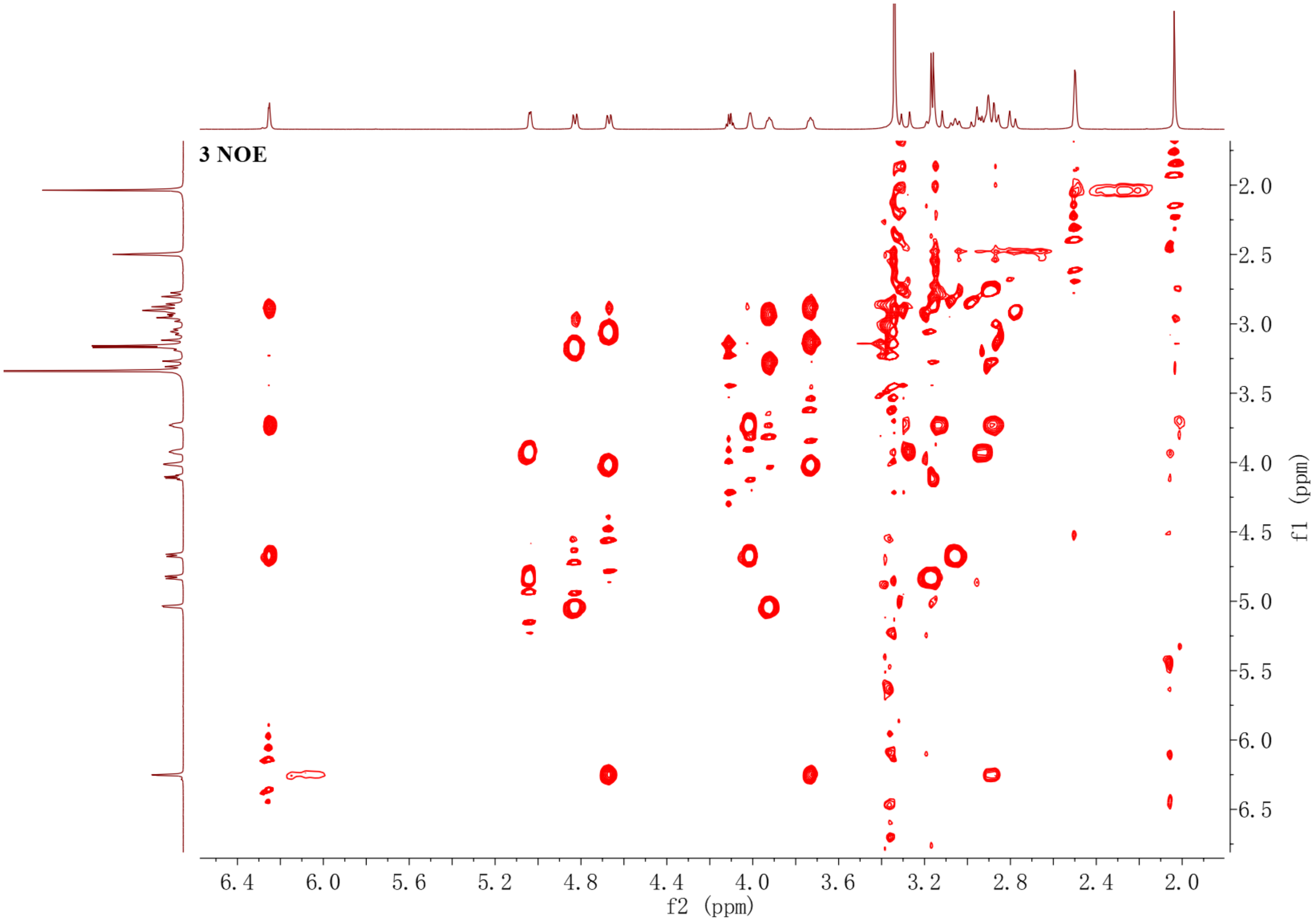

Figure S21. HR-ESI-MS of compound **3**.

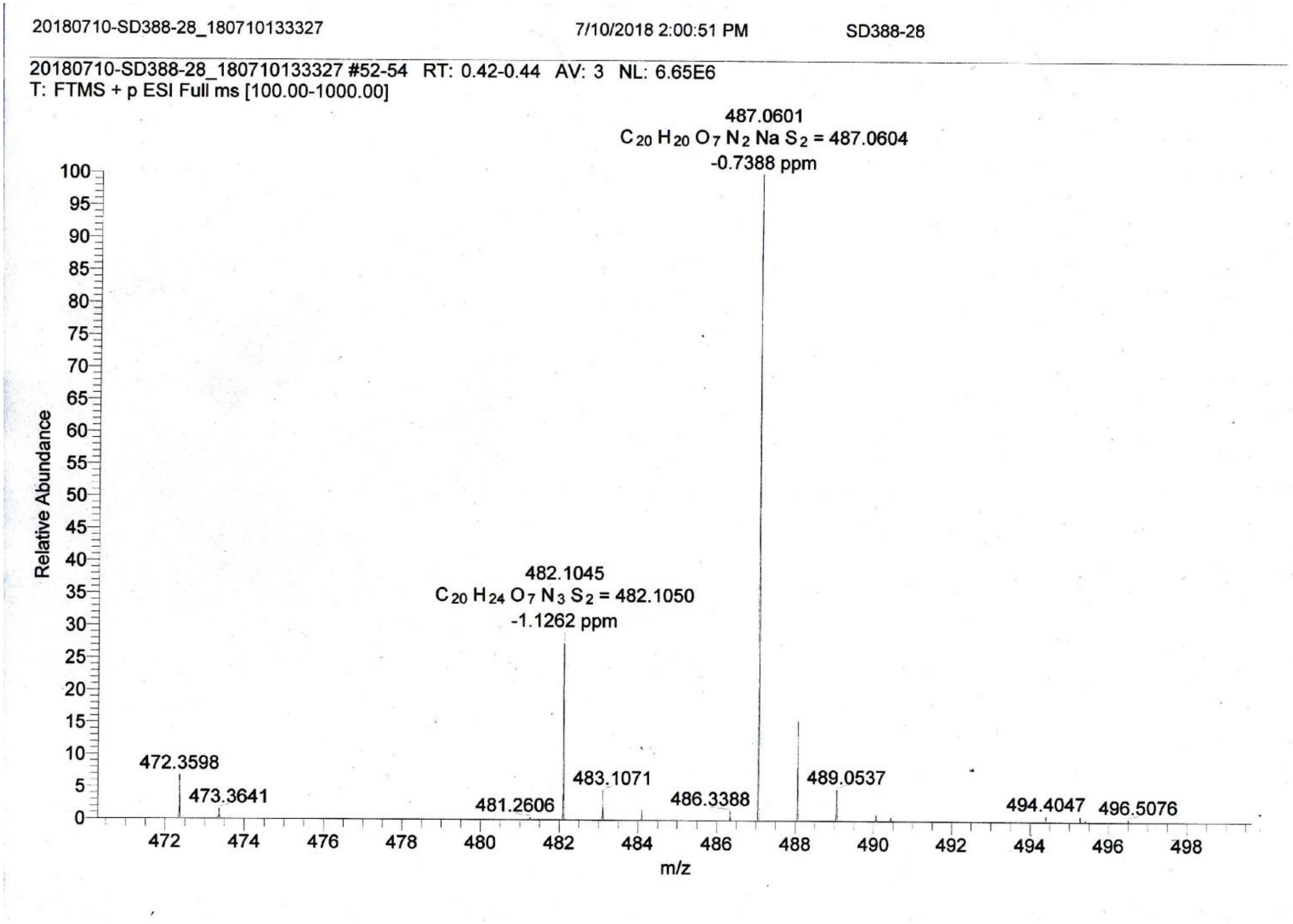

Figure S22. Crystal packing of compound **3** at 293(2) K;

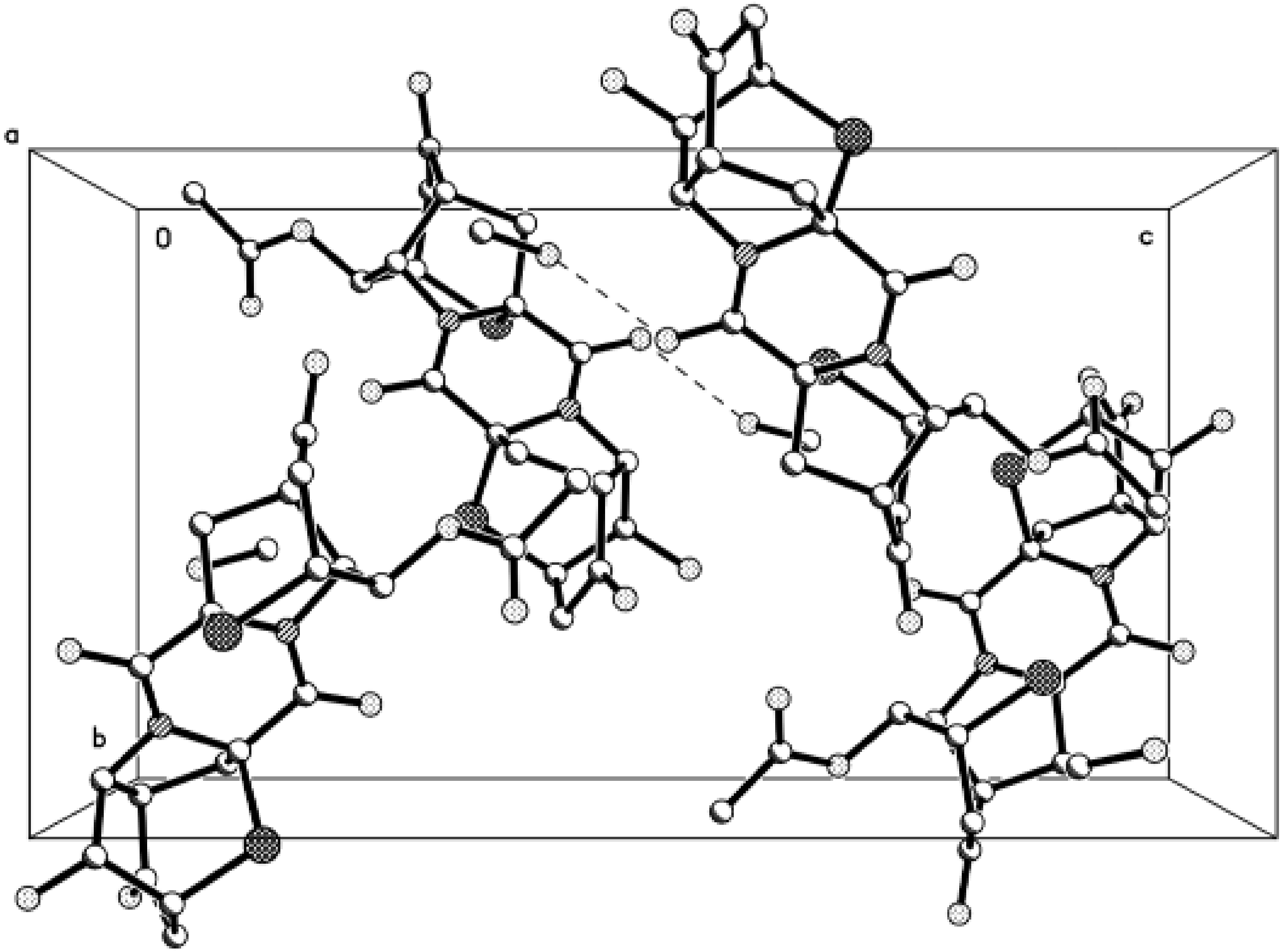

Figure S23. ^{1}H NMR (500 MHz, DMSO-d_6) spectrum of compound **4**.

4

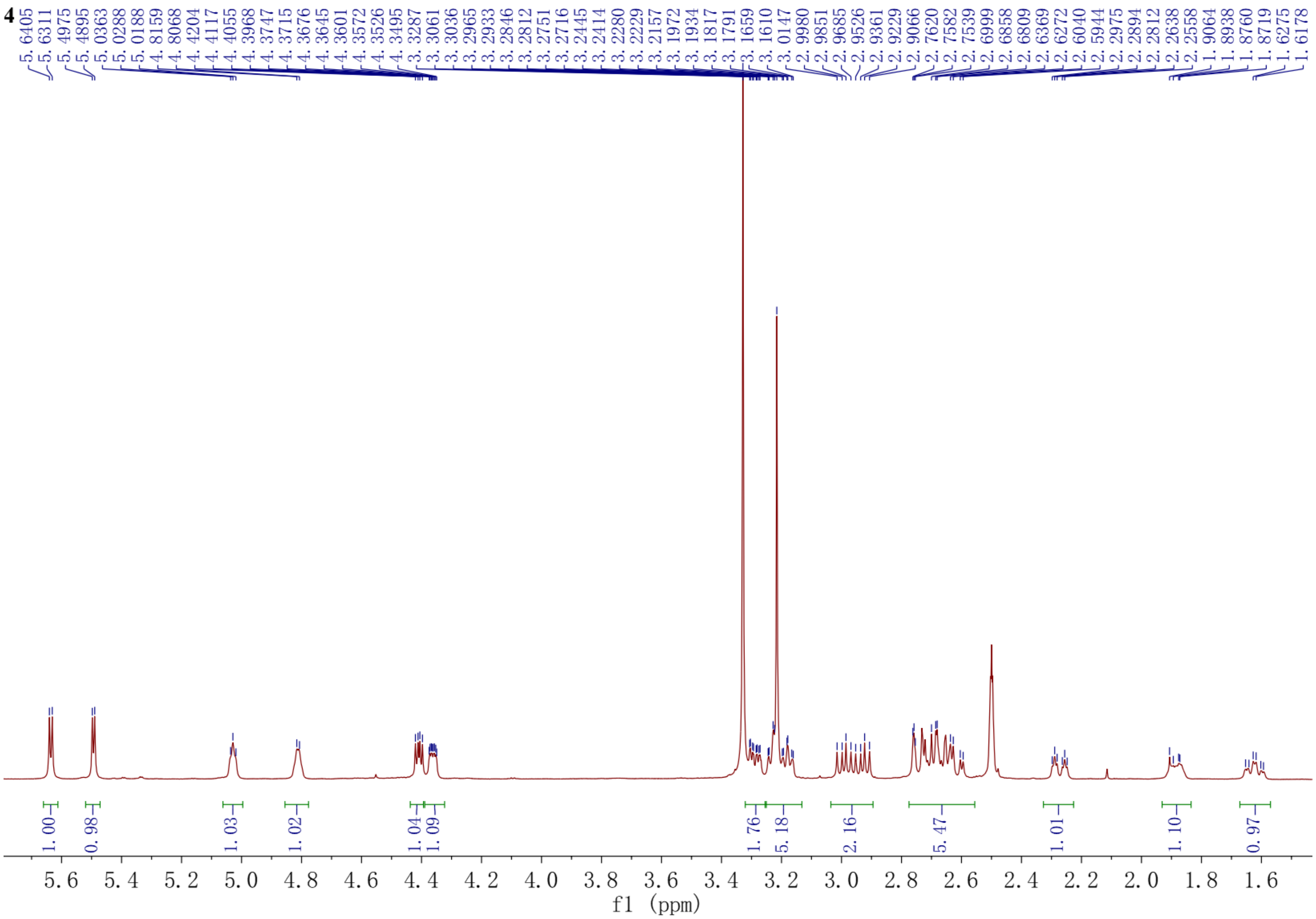

Figure S24. ^{13}C NMR and DEPT (125 MHz, DMSO-d_6) spectrum of compound **4**.

4

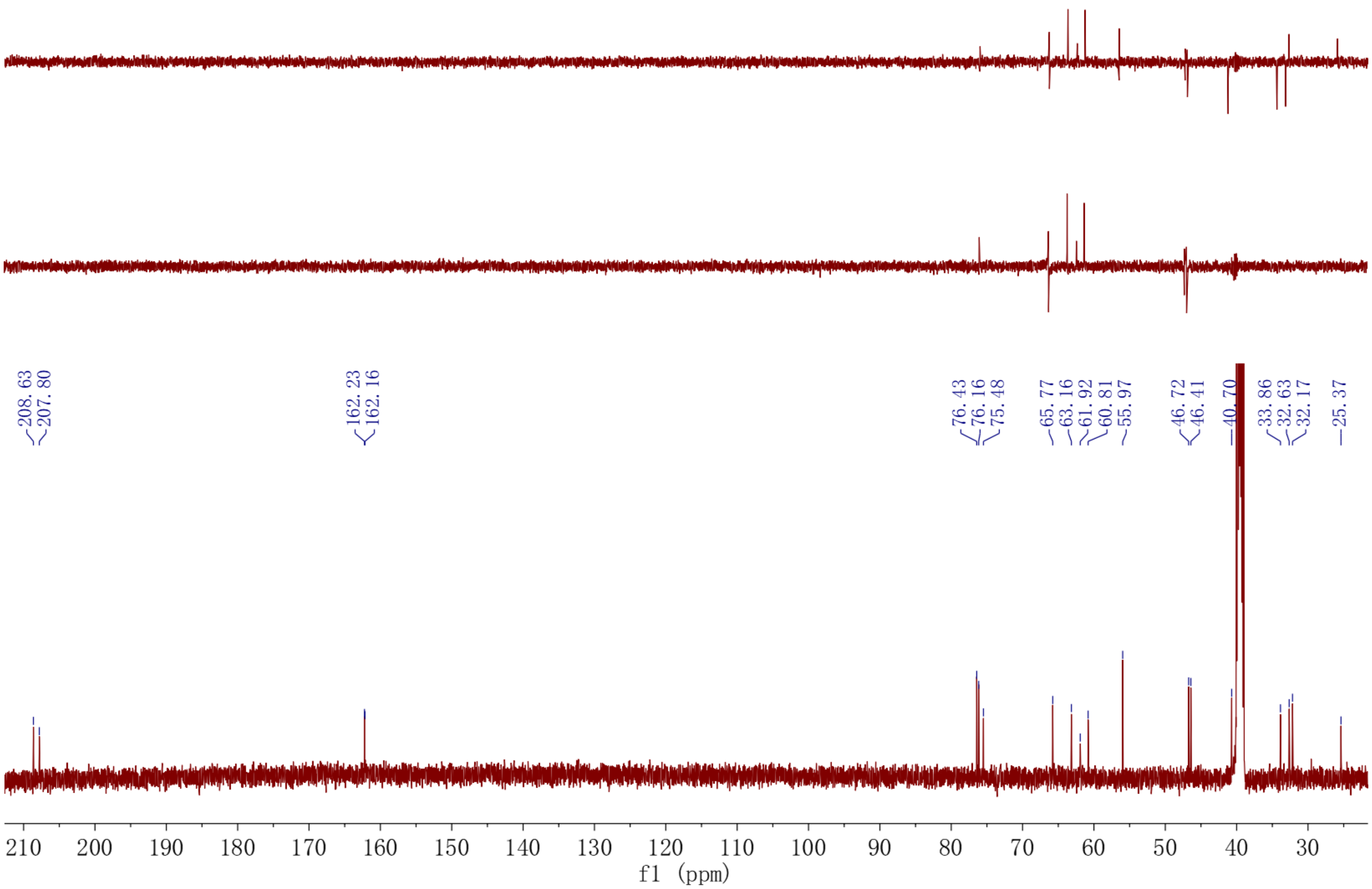

Figure S25. COSY spectrum of compound **4**.

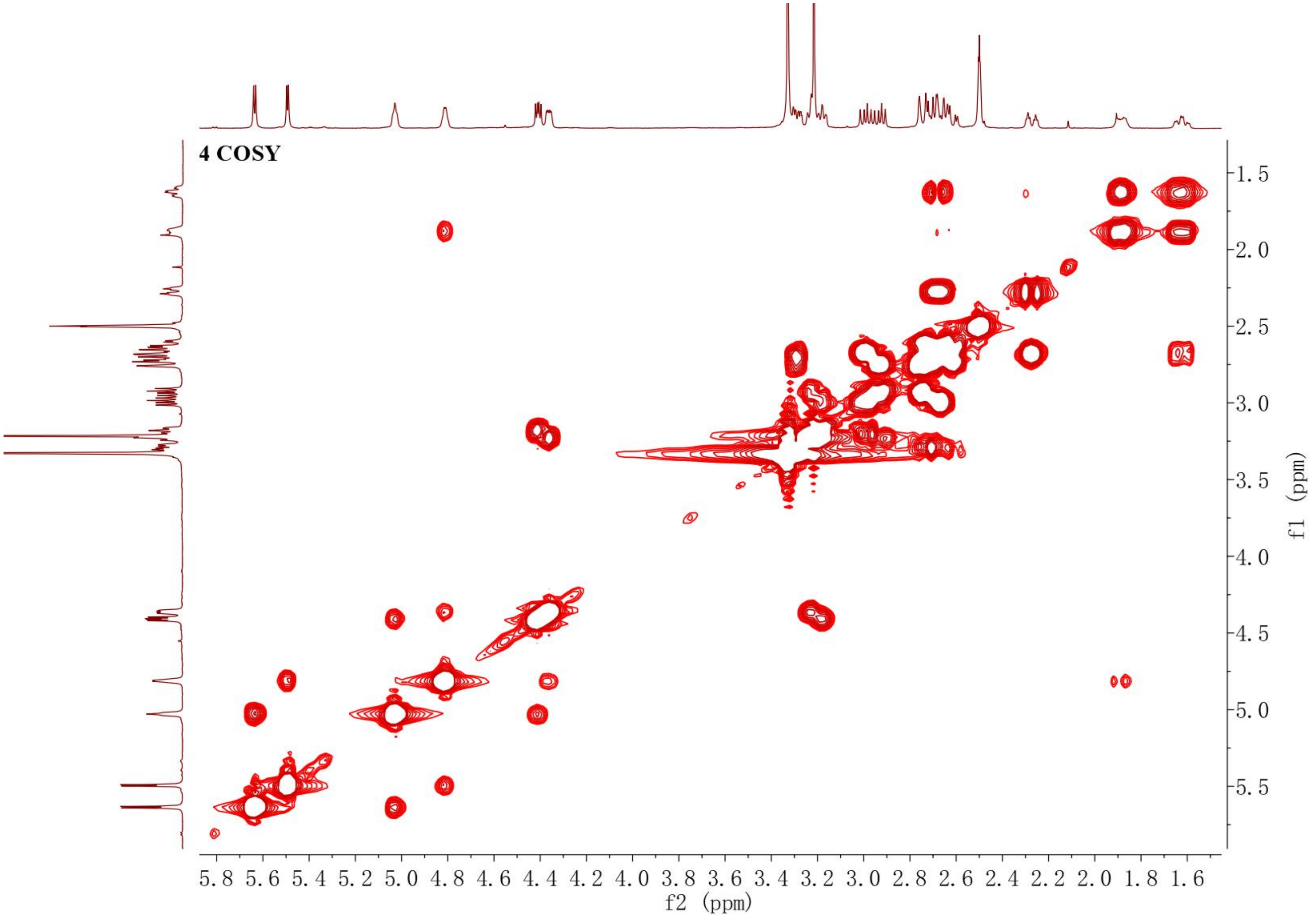

Figure S26. HSQC spectrum of compound **4**.

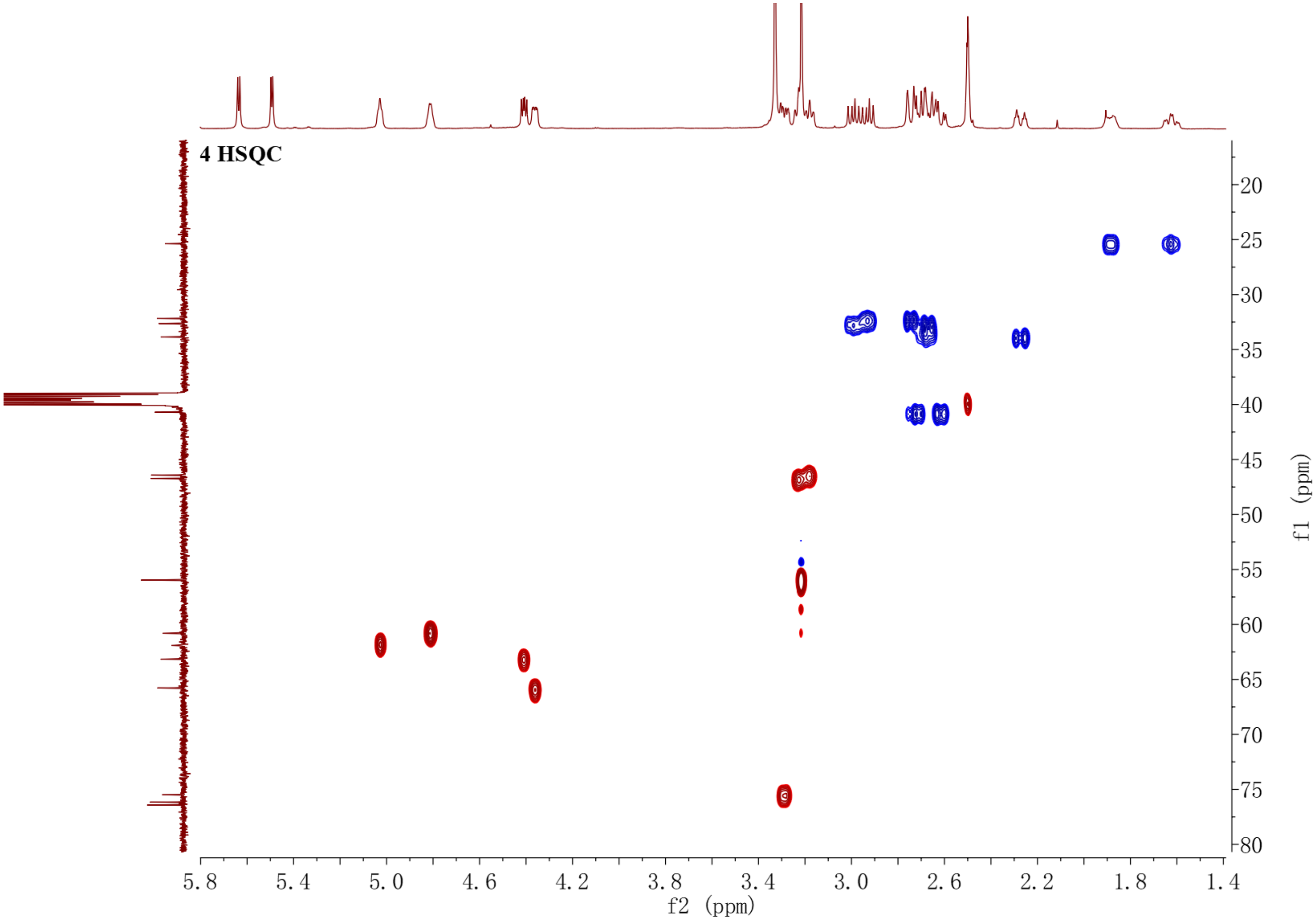

Figure S27. HMBC spectrum of compound **4**.

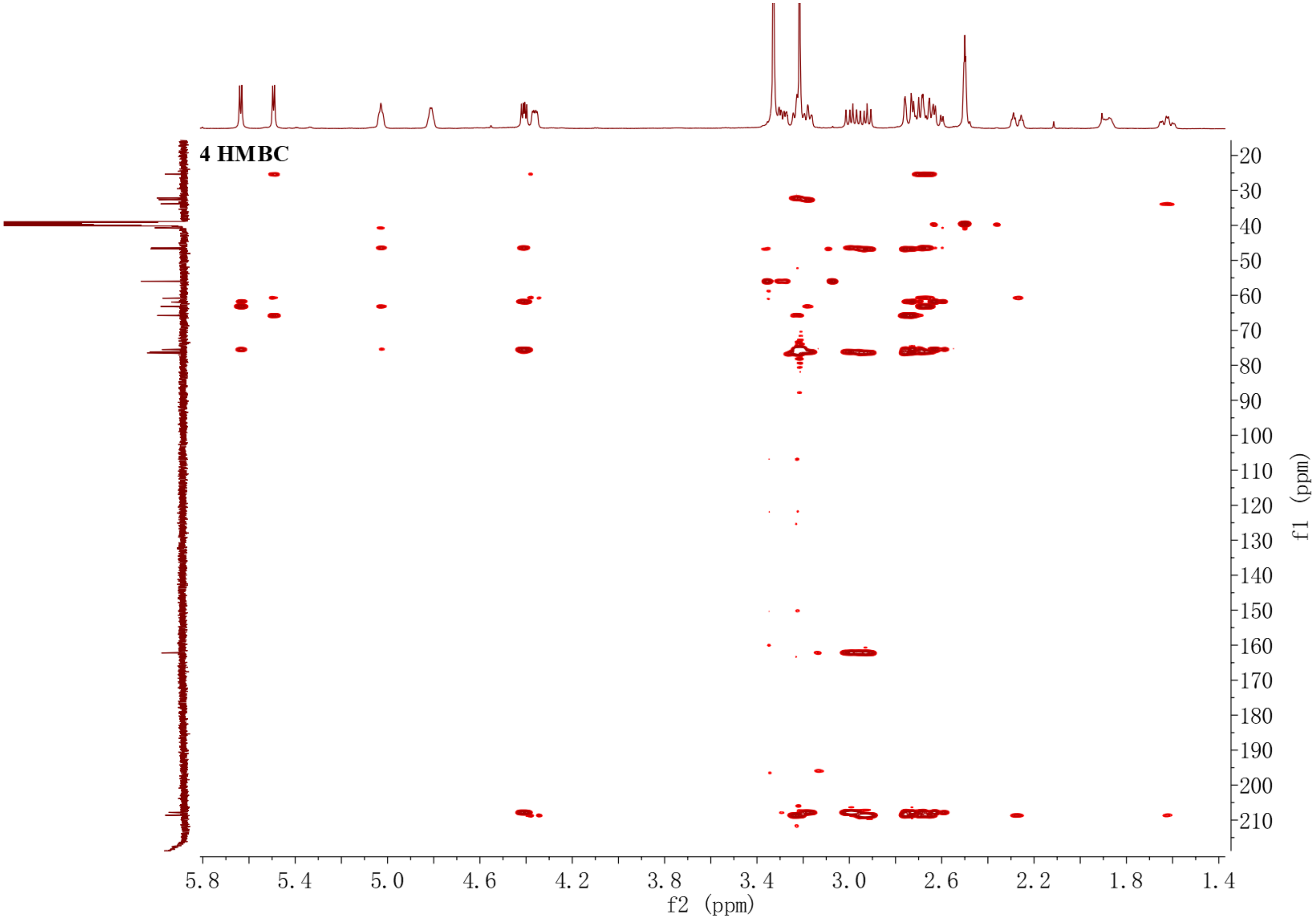

Figure S28. NOESY spectrum of compound **4**.

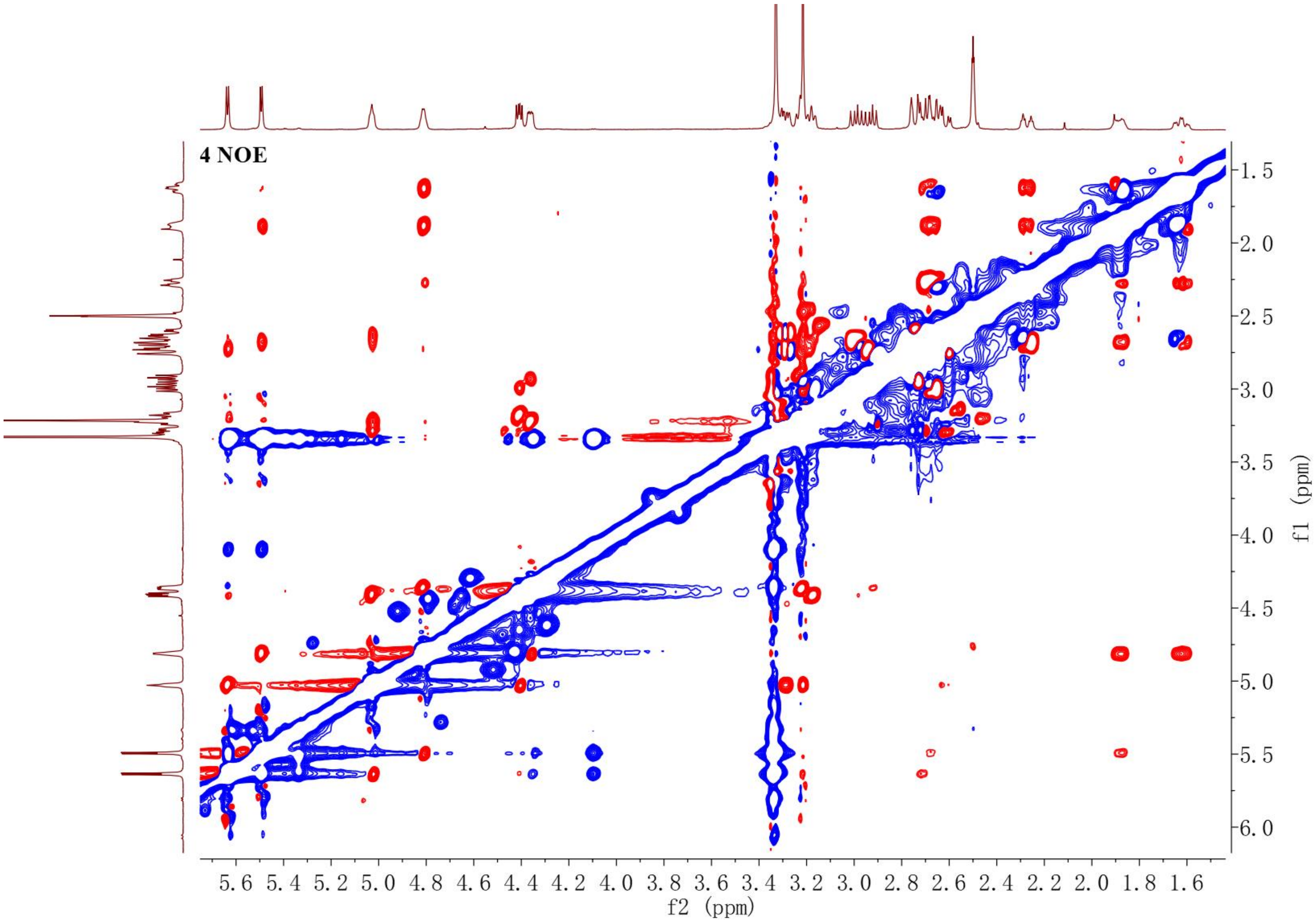

Figure S29. HR-ESI-MS of compound **4**.

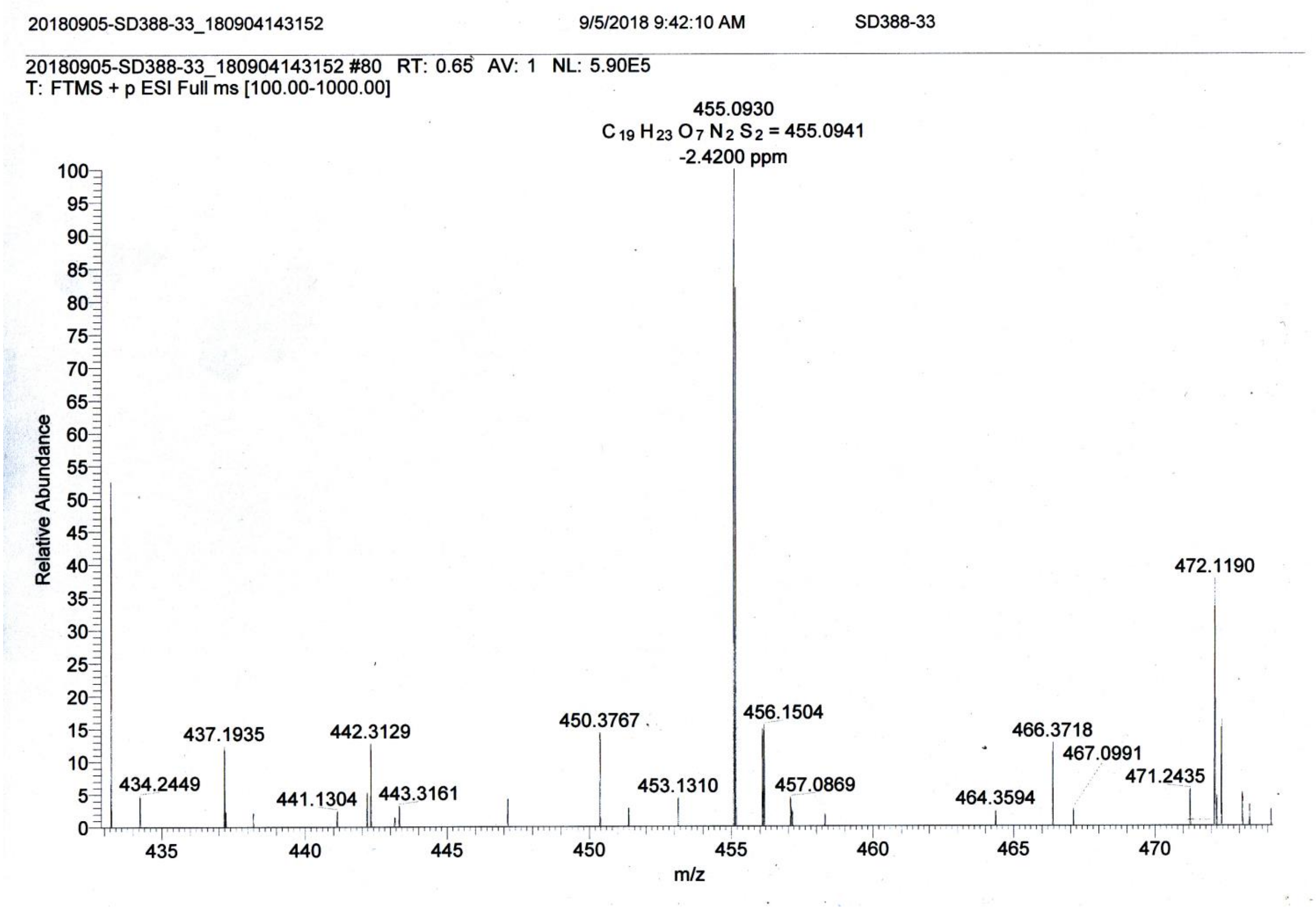

Figure S30. ECD spectrum of compound **4**.

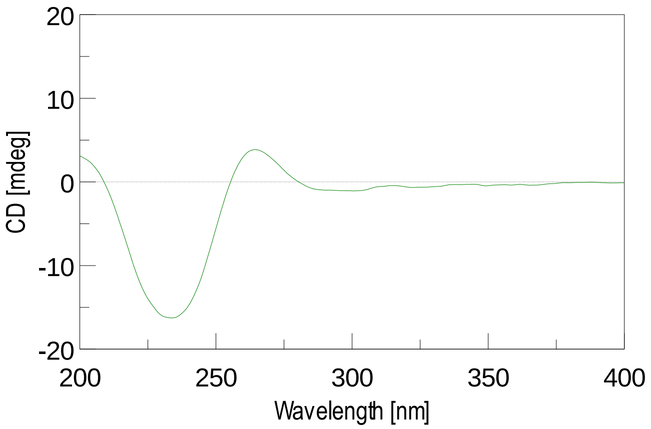

Figure S31. ^{1}H NMR (500 MHz, DMSO-d_6) spectrum of compound **5**.

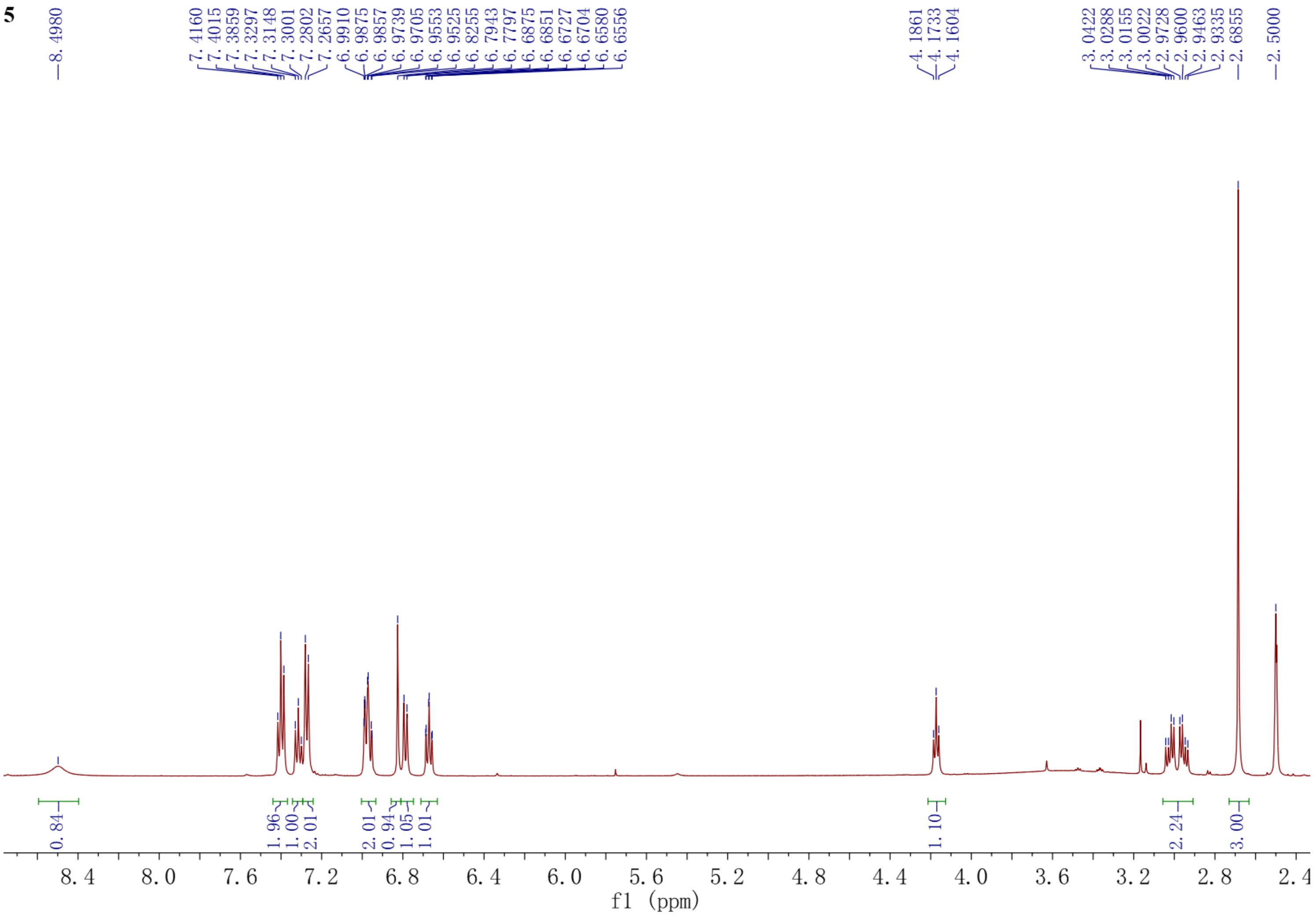

Figure S32. ^{13}C NMR and DEPT (125 MHz, DMSO-d_6) spectrum of compound **5**.

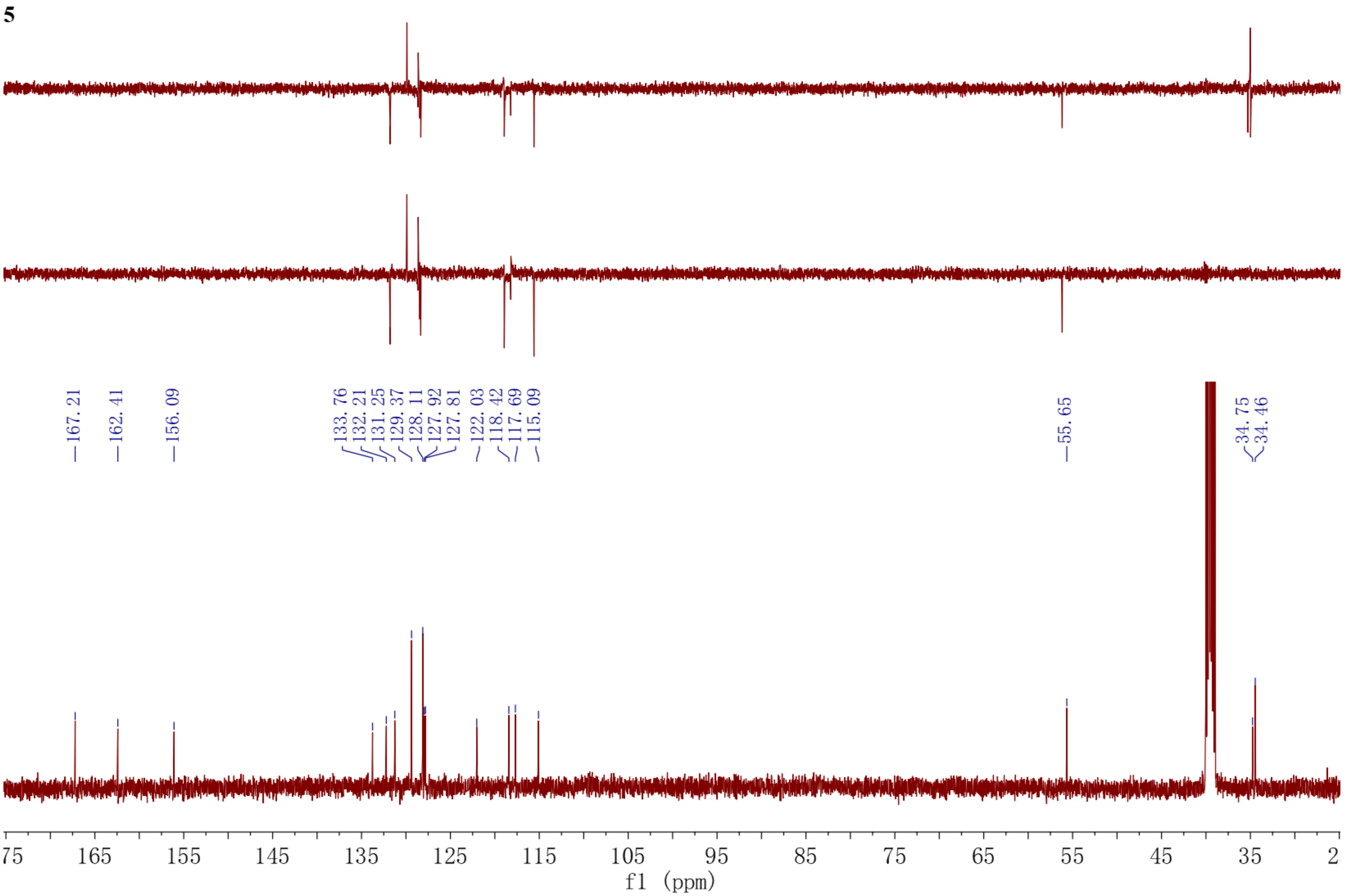

Figure S33. COSY spectrum of compound **5**.

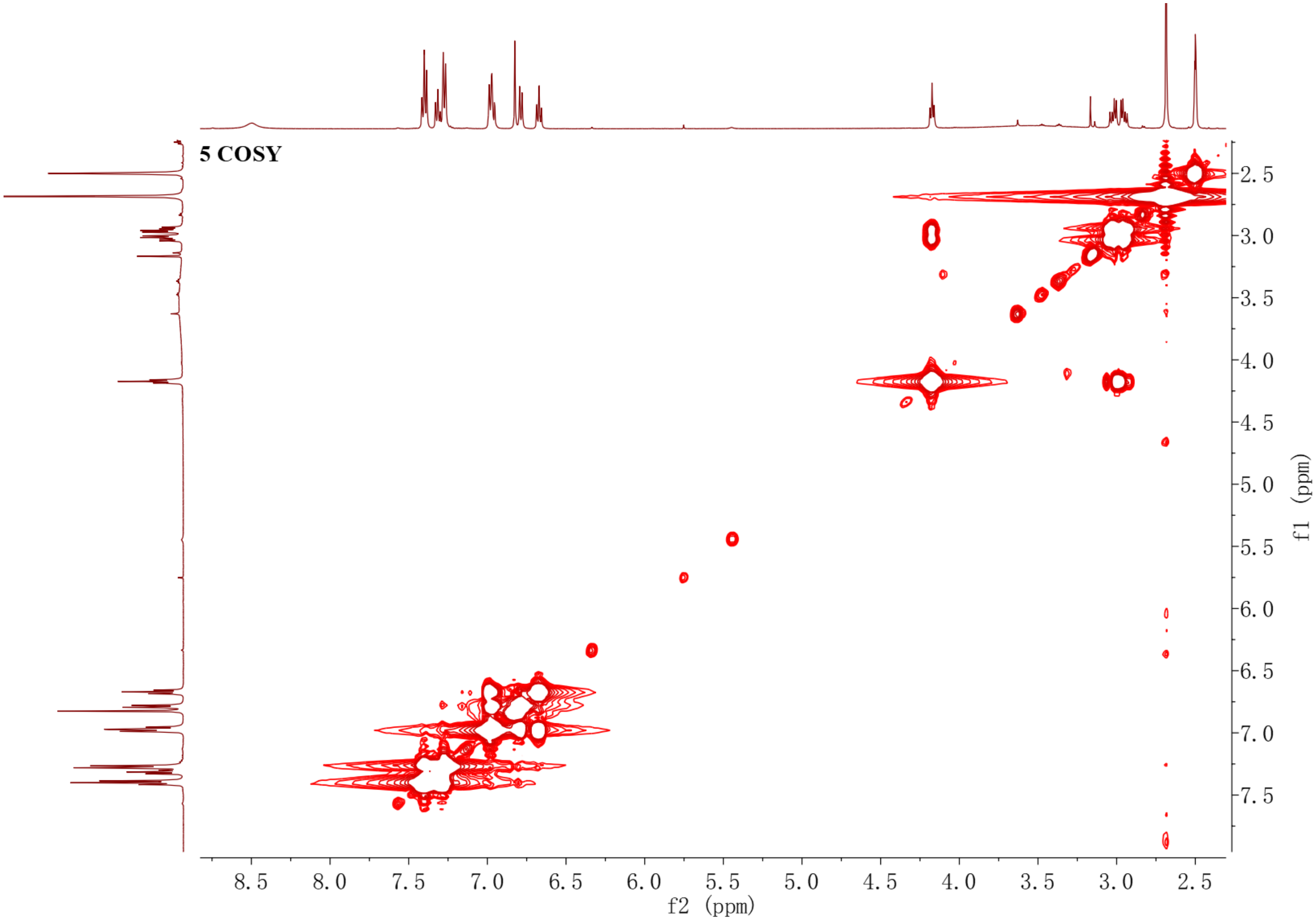

Figure S34. HMBC spectrum of compound **5**.

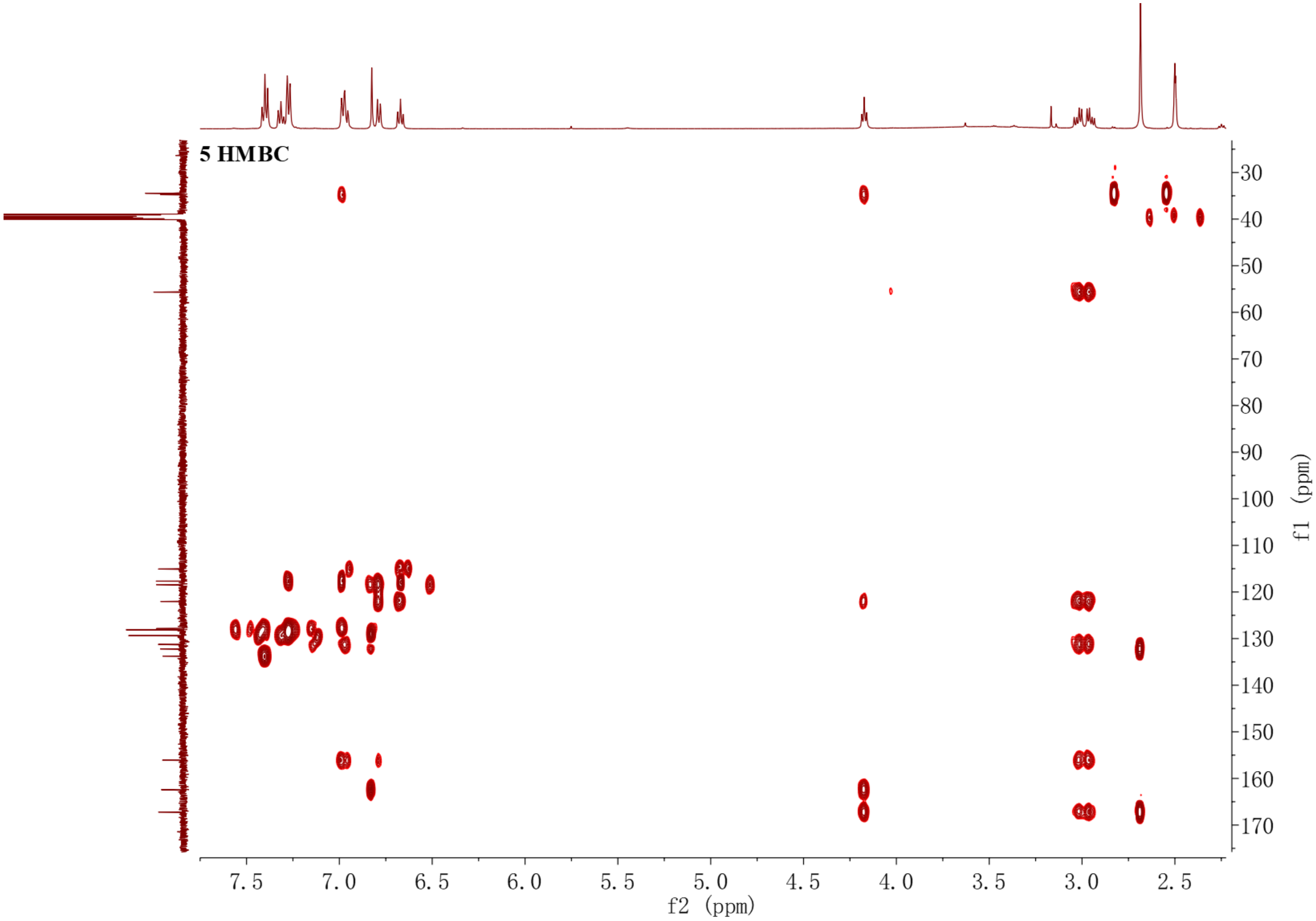

Figure S35. HR-ESI-MS of compound **5**.

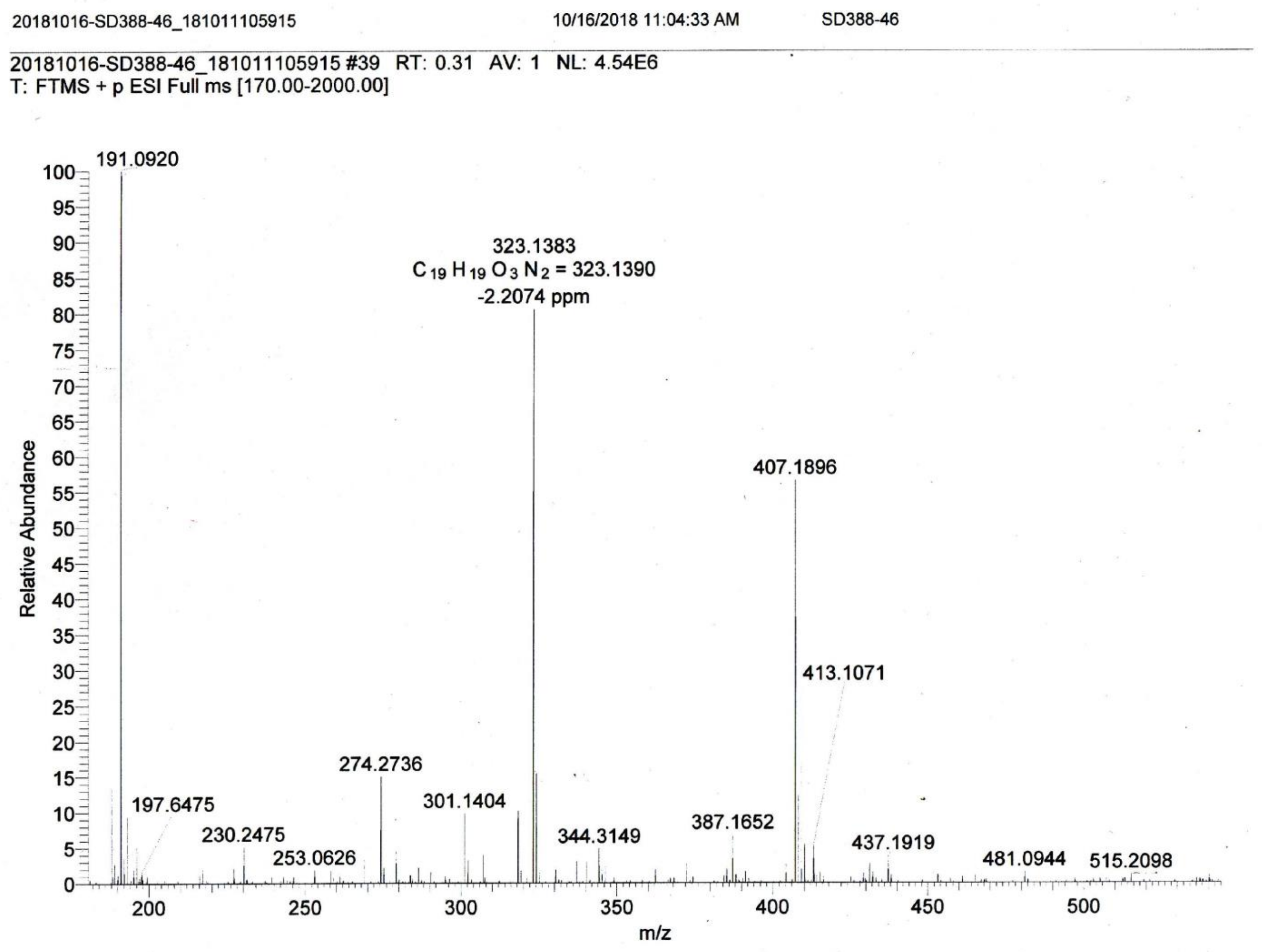

Figure S36. ECD spectrum of compound (±)-**5**.

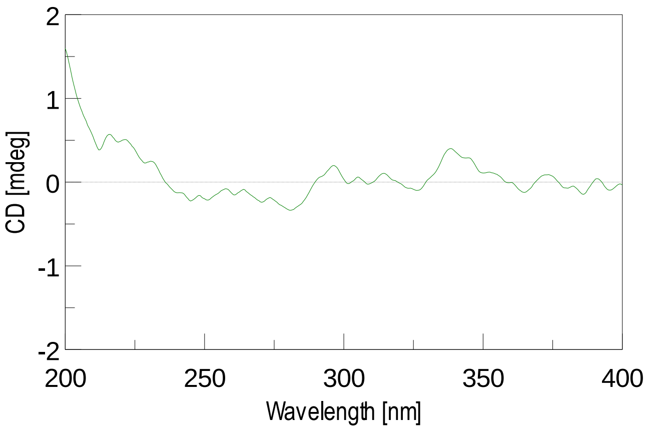

Figure S37. Chiral HPLC profile of compound (±)-**5**.

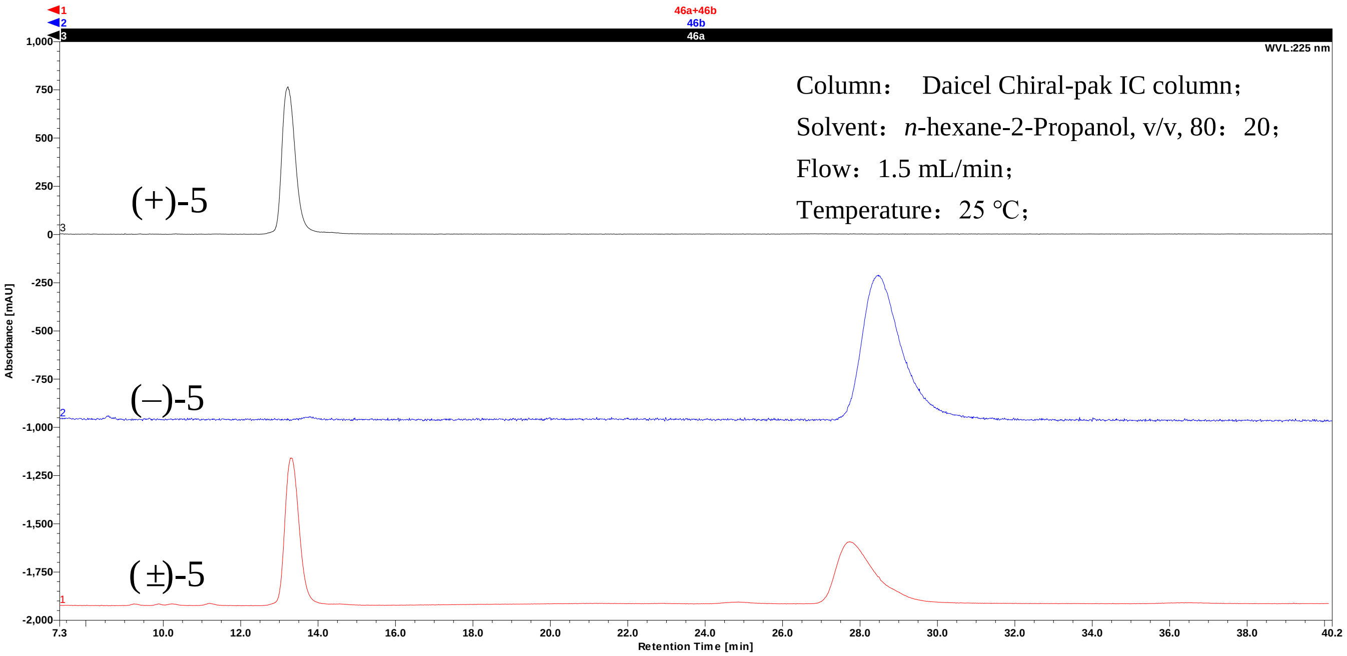

Figure S38. ECD spectrum of compound (+)-**5**.

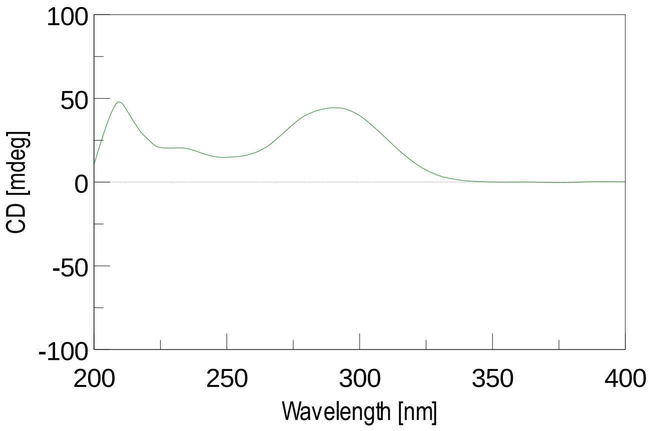

Figure S39. ECD spectrum of compound (–)-**5**.

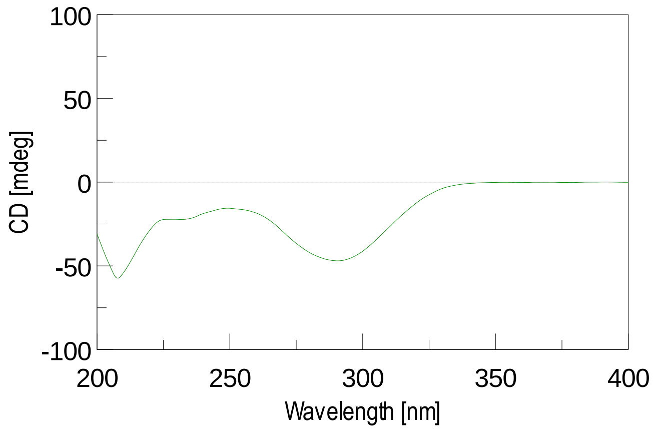

Supplement: Supplementary file 1 [file marinedrugs-18-00160-s001.pdf]
